# Quantifying the three-dimensional facial morphology of the laboratory rat with a focus on the vibrissae

**DOI:** 10.1371/journal.pone.0194981

**Published:** 2018-04-05

**Authors:** Hayley M. Belli, Chris S. Bresee, Matthew M. Graff, Mitra J. Z. Hartmann

**Affiliations:** 1 Department of Biomedical Engineering, Northwestern University, Evanston, Illinois, United States of America; 2 Northwestern University Interdepartmental Neuroscience Program, Northwestern University, Evanston, Illinois, United States of America; 3 Department of Mechanical Engineering, Northwestern University, Evanston, Illinois, United States of America; Northeastern University, UNITED STATES

## Abstract

The morphology of an animal’s face will have large effects on the sensory information it can acquire. Here we quantify the arrangement of cranial sensory structures of the rat, with special emphasis on the mystacial vibrissae (whiskers). Nearly all mammals have vibrissae, which are generally arranged in rows and columns across the face. The vibrissae serve a wide variety of important behavioral functions, including navigation, climbing, wake following, anemotaxis, and social interactions. To date, however, there are few studies that compare the morphology of vibrissal arrays across species, or that describe the arrangement of the vibrissae relative to other facial sensory structures. The few studies that do exist have exploited the whiskers’ grid-like arrangement to quantify array morphology in terms of row and column identity. However, relying on whisker identity poses a challenge for comparative research because different species have different numbers and arrangements of whiskers. The present work introduces an approach to quantify vibrissal array morphology regardless of the number of rows and columns, and to quantify the array’s location relative to other sensory structures. We use the three-dimensional locations of the whisker basepoints as fundamental parameters to generate equations describing the length, curvature, and orientation of each whisker. Results show that in the rat, whisker length varies exponentially across the array, and that a hard limit on intrinsic curvature constrains the whisker height-to-length ratio. Whiskers are oriented to “fan out” approximately equally in dorsal-ventral and rostral-caudal directions. Quantifying positions of the other sensory structures relative to the whisker basepoints shows remarkable alignment to the somatosensory cortical homunculus, an alignment that would not occur for other choices of coordinate systems (e.g., centered on the midpoint of the eyes). We anticipate that the quantification of facial sensory structures, including the vibrissae, will ultimately enable cross-species comparisons of multi-modal sensing volumes.

## Introduction

Facial anatomy, and particularly the anatomy of sensory accessory structures, has a long history of study. Many cranial sensory structures have been investigated in the context of psychophysics, biomechanics, and neuroanatomy, from the pinnae [1], to the eye [2–4], the nostrils [5], and the teeth [6–7]. In addition, animal skulls and muscles have been the subject of extensive morphometric analyses [8–14] with the semicircular canals receiving considerable attention [15–16].

Although these earlier studies provide a rich description of facial and sensory anatomy, few studies have examined the spatial relationships amongst the sensory structures. For example, the positions of a rat’s eyes relative to its mouth have not been compared with the corresponding relative positions in the cat. This knowledge gap limits our ability to determine how various animals differ in their acquisition of multi-modal sensory information as they navigate their environment.

The goal of the present work is to develop an approach towards quantifying the morphology of facial sensory structures in a manner that facilitates cross-species comparisons. This goal leads to an interesting problem: although all mammals have two eyes, two ears, two nostrils, and a single mouth, they can have very different numbers and arrangements of facial vibrissae (whiskers). Only mammals have true vibrissae, but vibrissal-like structures have evolved at least twice in vertebrates: in the mammalian lineage, as hair [17], and also in the avian lineage, as feathers, with birds showing a similar diversity of facial tactile feather arrangement [18–19]. This convergent evolution underscores the importance of this tactile sensory modality, and also highlights the need to characterize the morphology of these diverse sensory accessory structures.

The mammalian vibrissae are exquisitely sensitive tactile sensors, generally arranged in an array of rows and columns across the face [20]. The whiskers serve a multitude of behavioral functions across species, including navigation and climbing [21–22], wake following [23–24], anemotaxis [25], foraging [26–27], predation [28–29], and social interactions [30]. However, all previous studies that have examined the arrangement of whiskers on the face have quantified array morphology in terms of the discretized row and column positions of the whiskers [31–33]. A quantification approach based on discretized rows and columns is clearly incompatible with the need for cross-species comparisons.

In the present work, we first consider several possible choices for coordinate systems in which to quantify facial morphology and then deliberately choose “whisker-centric” axes based on the three-dimensional (3D) positions of the whisker basepoints. This choice allows us to quantify the morphology of the vibrissal array as well as the arrangement of other facial features and skull landmarks in terms of the whisker basepoint coordinates.

We anticipate that cross-species comparisons of the morphology of cranial sensory structures may lead to insights into the evolution and physiology of animal senses, thereby illuminating selection pressures within particular environmental niches that resulted in a variety of animal adaptations [34–36]. The present work begins what we anticipate will be a series of studies that quantify the 3D spatial relationships between whiskers, rhinarium, incisors, pinnae, mouth, and eyes across multiple species.

## Methods

All procedures were approved in advance by the Animal Care and Use Committee of Northwestern University.

### Data collection

Two Datasets containing a total of 518 macrovibrissae whiskers from nine rats were used. All animals were female Long Evans rats between the ages of 5 and 36 months.

#### Dataset 1

We reanalyzed data from 167 whiskers from three of the six rats used in the study of Towal et al., 2011. These three rats were first scanned in a three dimensional (3D) volumetric scanner. Then each whisker was plucked from the animal and scanned in 2D on a flatbed scanner. Of the 167 whiskers, 158 2D scans were analyzed to obtain measurements of arc length, and 130 were analyzed to obtain measurements of intrinsic curvature. The whiskers from the other three rats from Towal et al., 2011 were scanned only in 2D and were not reanalyzed in the present study.

Details of the methods for whisker acquisition are provided in Towal et al., 2011, but to summarize, euthanized rats were placed in a 3D laser scanner (Surveyor DS-3040) yielding a finely digitized 3D point cloud. The point cloud was imported into the software package RAPIDFORM XOR, where data points corresponding to each macrovibrissa were manually extracted from the point cloud. Manual rotation of the 3D scanned image was used to visually determine the set of points that clearly belonged to each whisker through all angles of rotation. Whisker basepoints were identified as the centroid of a small number of points (typically 8–20) on the mystacial pad that rotated the least relative to that identified macrovibrissa. A moving average (21-sample window) was used to smooth the shape of each macrovibrissa, finally yielding a set of 3D (x, y, z) points along each whisker length for each of the three rats. These same whiskers, from both right and left sides of the animal, were plucked from the rat and scanned in 2D on a flatbed scanner (Epson Perfection 4180 Photo) at a resolution of 2,400 dpi (10.6 microns per pixel) to quantify whisker arc length and curvature. No skull or facial features were acquired from the rats in Dataset 1.

#### Dataset 2

**Fig 1:**
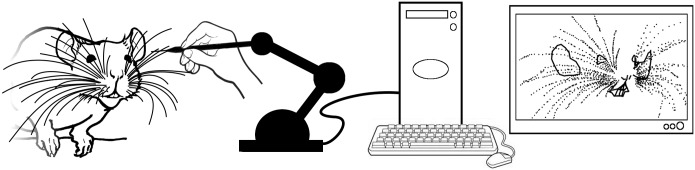
Schematic depicting the data collection process for Dataset 2. The shapes of the whiskers and facial features of anesthetized rats were manually traced using a Microscribe^™^ 3D Digitizer. These 3D traces were imported into Matlab^™^ and placed in standard orientation and position.

A schematic of the data collection process for Dataset 2 is shown in Fig 1. A total of 351 whiskers were acquired from the left and right arrays of six rats using the following methods.

#### Anesthesia and surgery

Animals were anesthetized with an intraperitoneal injection of a ketamine/xylazine mixture (58.3 mg/kg ketamine hydrochloride, 2.92 mg/kg xylazine hydrochloride, and 0.58 mg/kg acepromazine maleate in a saline vehicle). A deep plane of anesthesia was maintained throughout the procedure by monitoring for the toe pinch withdrawal reflex every fifteen minutes and administering booster doses of anesthetic as needed. The animal was placed on a heating pad and secured in a stereotaxic apparatus including a bite block and ear bars. The stereotaxic unit ensured that the animal’s head, neck, and forepaws were suspended in air and no vibrissae touched the heating pad. An incision was made along the scalp midline, and three skull screws were secured to form a triangle, with one screw in the frontal bone and one in each temporal bone. A bridge of methyl methacrylate (dental acrylic) was then constructed between the arm of the stereotaxic and the skull screws, taking care to leave the skull landmarks lambda and bregma exposed when possible [37]. Once the acrylic had cured and the animal’s head was stable, the bite block and ear bars were removed, ensuring that no object touched any part of the animal’s face or vibrissae.

#### Data acquisition with the Microscribe^™^

Whiskers were traced using a G2X Microscribe^™^ 3D Digitizer (Revware). This device consists of a passively movable five degree-of-freedom mechanical arm with a probe of known length on the tip. A user manipulates the arm to place the tip of the probe at a location in space. The device reports (x, y, z) coordinates of this spatial location relative to a previously defined home location [0,0,0]. Manufacturer listed mean precision is 0.13 mm and mean accuracy is 0.23 mm, using a conical steel tip measuring 24.5 mm in length x 5.0 mm in diameter, with a slope of 0.5 (the default tip). This tip was too thick to maneuver easily between whiskers, so instead we used a 0.5 mm x 4.4 cm steel wire held in a pin vice (Starrett; 75.0 mm in length x 5.0 mm in diameter), custom machined to thread into the probe-holder of the Microscribe^™^ tip. The portion of the steel wire clamped in the vice was 2.4 cm long, and 2 cm of the steel wire was exposed. Manufacturer’s instructions for calibrating to the custom tip were followed prior to collecting data from each animal.

As schematized in Fig 1, the probe at the end of the Microscribe^™^ was held in the experimenter’s dominant hand, and the tip was sequentially placed at a series of points on each of the anatomical structures of interest. At each point, a button was pressed to record the 3D coordinates (x, y, z) representing the position of the Microscribe^™^ tip. For all six rats in Dataset 2, we digitized the basepoint of each whisker and multiple points along each whisker’s length. For four rats in Dataset 2, we digitized the skull features lambda and bregma. For five of the six rats in Dataset 2, we digitized points corresponding to the corners and contours of prominent facial features including the eyes, nostrils, rostrum, mouth, pinnae, and incisors.

#### Error assessment

At the start of each experiment we established an origin for the Microscribe^™^ by making a small divot in a piece of laboratory labeling tape on the operating table near the animal. We sampled this origin from ~20 different orientations to calibrate the device. Over all scans and experiments, we recorded a mean resolution of 0.5 mm. This resolution includes error due to any hand tremor of the user, any slight eccentricities of the tip, and the intrinsic precision limitations of the device. Note that, because each point along the whisker is an independent observation for that whisker, the 0.5 mm error does not compound along the length of the whisker.

To obtain an accurate estimate of measurement error for Dataset 2, we performed simulations in which we randomly varied all the points along the whisker length (including the basepoint) by +/- 0.5 mm in all three spatial dimensions, and observed the effect on the 3D position and orientation of the whiskers. We performed these simulations for two whiskers from each row (1 caudal, 1 rostral). The whisker basepoint locations were found to have a maximum error of 0.01 mm in r_bp_, 0.24° in θ_bp_, and 0.19° in φ_bp_, and the whisker emergence angles were found to have a maximum error of 4.8° in θ_w_, 3.0° in φ_w_, and 2.7° in ζ_w_. Each of these variables is defined in the sections below.

#### 2D whisker scanning

After data collection with the Microscribe^™^ was complete, animals were euthanized with an overdose of the ketamine-xylazine-acepromazine combination, followed by decapitation. Whiskers from four of the six rats were trimmed at the base using forceps and micro scissors, and stored in folded rectangles of aluminum foil for one to two days. The whiskers were then scanned, along with a calibration ruler (1 mm resolution), using a flatbed scanner (Epson Perfection 4180 Photo) at a resolution of 2,400 dpi (10.6 microns per pixel). A total of 244 whiskers were scanned in 2D for Dataset 2. Of those 244 whiskers, 226 were of high enough quality to obtain measurements of arc length, and 222 were of high enough quality to obtain measurements of intrinsic curvature.

### Definition and quantification of whisker morphological parameters

A total of eight parameters were used to quantify whisker array morphology. Two parameters described 2D whisker geometry: the arc length (S) and the intrinsic curvature coefficient (A). Three parameters described the 3D coordinates of the whisker basepoints (r_bp_, θ_bp_, φ_bp_), and three parameters described the Euler angles at which the whiskers emerged from the mystacial pad (θ_w_, φ_w_, ζ_w_).

#### Quantifying whisker arc length

Values for arc length for 158 of the 167 whiskers of Dataset 1 [33] were obtained from the original study. No new measurements for arc length were made for these rats.

Values for arc length for 226 of the 244 whiskers of Dataset 2 scanned in 2D were obtained by importing the scanned images into Matlab^™^. The whisker shape was extracted by manually clicking on 8–15 points along the whisker in the images. Those traces were upsampled to 100 points using cubic spline interpolation. The arc length was calculated by summing the lengths of the discretized segments that composed the trace. Measurement error was approximately two pixels on each end of the whisker, or ~42.4 microns (~10.6 microns per pixel x 2 pixels per endpoint x 2 endpoints).

It is important to note that the whiskers were plucked from the rats in Dataset 1 [33], while the whiskers were trimmed from the rats in Dataset 2. The average arc length values for Dataset 1 were therefore slightly larger than those for Dataset 2. To correct for this discrepancy, we performed a separate analysis of follicle morphology. We serially sectioned four mystacial pads from three animals (female, Long Evans rats, between three and eight months in age) and formed z-stacks of the sections to quantify the length of each follicle. For each whisker identity (e.g., the C3 whisker), we found the median length of the follicle across all four mystacial pads. We then subtracted this median length from the arc length reported for each whisker of Dataset 1.

To validate this approach, we grouped whiskers by their (row, column) identity and then plotted whisker arc length from Dataset 1 against whisker arc length from Dataset 2, before and after application of the follicle-length correction factor. The arc lengths of Dataset 1 were a better match to those in Dataset 2 after subtracting the median follicle length for each whisker (Wilcoxon signed rank test: p < 0.0084 before correction; p < 0.44 after correction).

#### Quantifying the intrinsic curvature coefficient

Previous studies have shown that the proximal ~60%-70% of the whisker lies in a plane, with a 2D shape that can be approximated by the parabola y = Ax^2^ [32–33]. However, the value for the intrinsic curvature coefficient (A) depends strongly on exactly how the base of the whisker is aligned with the x-axis.

In the present work, we aimed to reduce variability in the alignment of the proximal portion of the whisker. The whisker basepoint was first placed at the origin (0,0) and the whisker was oriented concave up along the x-axis such that the majority of the whisker lay in the first quadrant. The whisker was then truncated to 65% of its total arc length. To determine what fraction of the truncated whisker to align with the x-axis, we performed an optimization routine that iteratively rotated the whisker such that between 1 and 30% of the proximal portion of the truncated arc length was aligned with the x-axis. The curve y = Ax^2^ was then fit to the truncated whisker at each of these rotations. The mean squared error (MSE) was calculated between the actual smoothed whisker trace and the curve y = Ax^2^ at each of the alignments. When averaged across all truncated whiskers, error was minimized by aligning at a point 8% out along the truncated whisker length from the base. All whiskers were therefore aligned with the x-axis at a point 8% out along their truncated length from the base.

#### Choice of axis conventions in which to analyze the morphology of the array

Before we could define the 3D coordinates of the whisker basepoints (r_bp_, θ_bp_, φ_bp_) and the Euler angles (θ_w_, φ_w_, ζ_w_) at which the whiskers emerged from the mystacial pad, we first needed to choose an origin and horizontal plane in which to orient the rat’s head.

**Fig 2:**
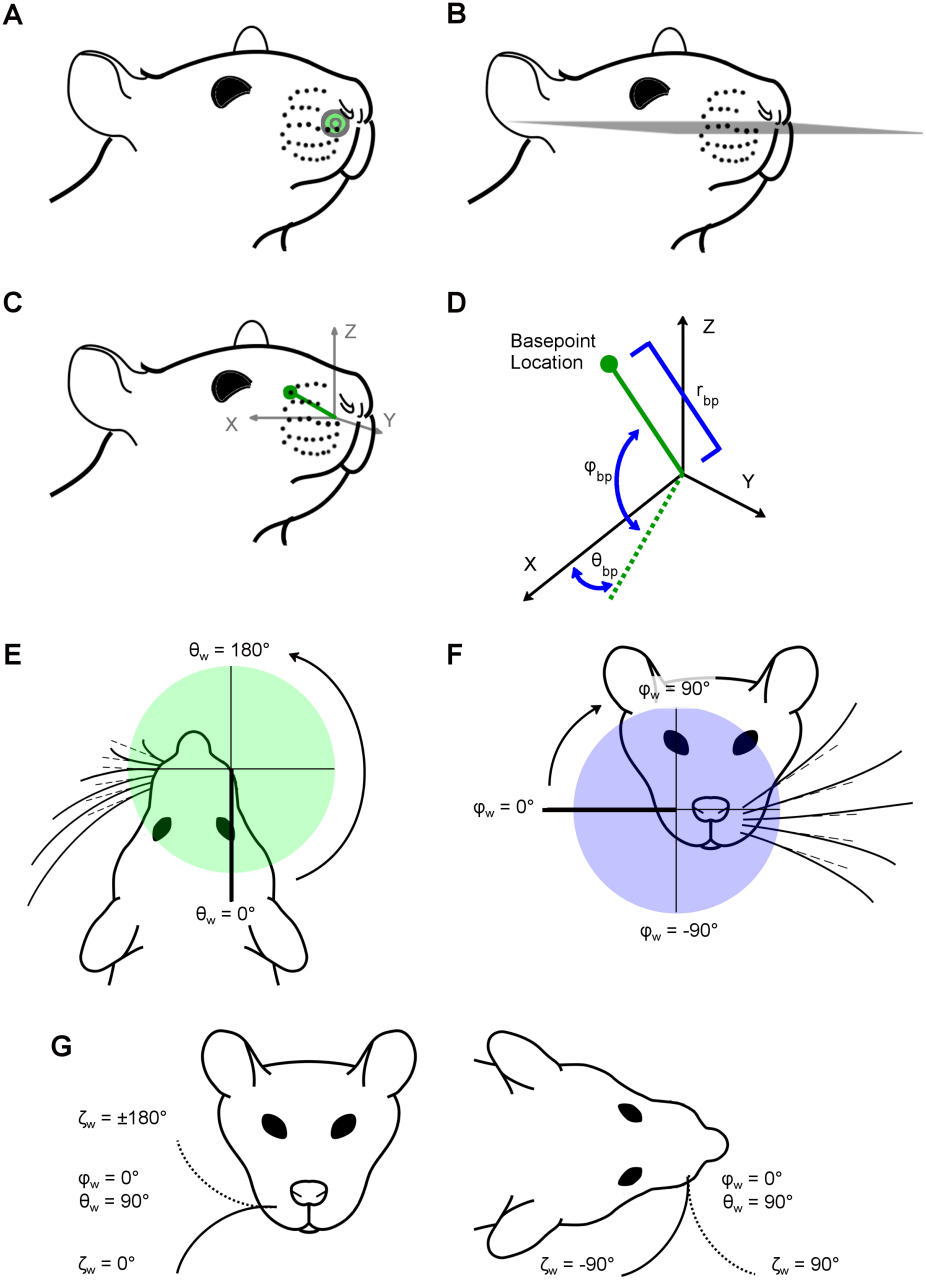
Definitions of whisker basepoint coordinates and angles of emergence. Subplots A-D describe whisker basepoint coordinates, while subplots E-G describe whisker emergence position and orientation. (A) The origin (green dot) is defined as the average of all left and right whisker basepoint locations. The origin is not on the surface of the snout, but is the bilateral center of the array. (B) The horizontal plane is defined by the average whisker row plane, i.e., the mean of the five planes fit individually to each of the five whisker rows. (C) Axis conventions in the context of the head, illustrating that the origin is at the mean location of all whisker basepoints and the centroid of the right array basepoints lies along the positive x-axis. (D) A schematic of the axis conventions used to describe basepoint coordinates shows how the radius (r_bp_), azimuth angle (θ_bp_), and elevation angle (φ_bp_) are measured. The angle θ_bp_ is defined in the x-y plane from -90° (caudal) to +90° (rostral), where θ_bp_ = 0° lies along the x-z plane. The angle φ_bp_ is defined as the signed angle between the basepoint’s position vector (connecting the origin to the basepoint location) and the x-y plane, from -90° (ventral) to +90° (dorsal), where φ_bp_ = 0° lies along the x-y plane. (E) Top-down (horizontal) view of the rat face, illustrating the axis conventions in which θ_w_ is defined. Dashed lines represent the vector aligned with the proximal, approximately linear portion of the whisker. The angle θ_w_ describes the rostral/caudal angle at which the whisker emerges from the mystacial pad. Values range from 0° to 360°, where θ_w_ = 0° lies along the negative y-axis. The value of θ_w_ is independent of the intrinsic curvature of the whisker. (F) Front-on (coronal) view of the rat face, illustrating the axis conventions in which φ_w_ is defined. The angle φ_w_ describes the dorsal/ventral angle at which the proximal portion of the whisker emerges from the mystacial pad. Values range from -90° to 90°, and φ_w_ = 0° lies along the positive x-axis. (G) Schematics showing front-on and top-down views of the rat face, illustrating the axis conventions in which ζ_w_ is defined. The angle ζ_w_ describes the orientation of the whisker about its own axis. Solid and dashed lines represent extreme positions of the whisker in each view. ζ_w_ is the rotation of the whisker around its own axis. This subplot illustrates ζ_w_ for the case that θ_w_ = 90° and φ_w_ = 0° in order to show the whisker in a more naturalistic position. ζ_w_ = 0° points concave down, ζ_w_ = 90° concave forward, ζ_w_ = -90° concave back, and ζ_w_ = 180° concave up.

In the present work, we chose the origin (0,0,0) to be the mean position of all whisker basepoint locations on both left and right sides of the array. This placed the origin inside the animal’s head, near the center of the muzzle (Fig 2A). Note that this procedure required “matched” basepoints between right and left sides; in the case that a basepoint for a particular whisker identity was missing on one side, its non-missing complement was omitted from the calculation of the origin.

Following the approach used in Towal et al., 2011, we used the “average whisker row plane” as the horizontal (x-y) plane. Planes were fit to the basepoints of the combined left and right whisker rows using least squares, producing one plane for each of the five whisker rows A–E. The mean of these five planes defined the average whisker row plane (Fig 2B).

From the average whisker row plane, axis conventions were established (Fig 2C). The x-axis was defined by connecting the centroid of the whisker basepoints on the right side of the face with the centroid of the whisker basepoints on the left side of the face. The center of the left array lay along the negative x-axis and the center of the right array along the positive x-axis. The rat head was then pitched about the x-axis such that the average whisker row plane defined the x-y plane. The y-axis pointed rostrally, and the negative y-axis pointed caudally. Finally, the z-axis was defined to be perpendicular to the x-y plane.

#### Quantifying 3D basepoint coordinates of the whiskers

Having chosen an origin and horizontal plane, the 3D coordinates of all recorded points on the animal’s head, including the whisker basepoints, could be determined.

Previous studies have used a standard spherical coordinate system to define the positions of the whisker basepoints [33]. The advantage of this system is that it is mathematically consistent in a way that makes it easy to run simulations [38–42]. However, a major disadvantage of this choice is that the left and right arrays are described in the context of the whole head, not in terms of an individual array, i.e., corresponding whiskers on left and right sides have completely different coordinates. A conventional head-centered spherical coordinate system essentially ignores the bilateral symmetry of the head, treating left and right sides as qualitatively distinct. This description is counterintuitive if one wishes to describe a canonical array, whether on the left or right of an animal.

We therefore chose axis conventions that mirror the left array across the midline of the head (y-z plane), in effect describing it as a right array. Mirroring the full left array (basepoints and whiskers) was completed with the head placed in standard position and orientation, prior to any calculations being performed. This technique constructs an “array-centered” axis convention that describes right arrays. Therefore, all calculations, figures, and equations are presented in terms of right-sided whisker arrays. To generate a left array, for the purposes of a simulation, for example, the final step of generating an array shape would be to mirror the Cartesian coordinates back across the midline of the head.

As shown in Fig 2D, the coordinate θ_bp_ describes the rostro-caudal location of the basepoint with respect to the positive x-axis. A positive value of θ_bp_ indicates that the whisker is rostral to the x-z coronal plane (at the location y = 0), while a negative angle indicates that the whisker is caudal to this coronal plane. The coordinate φ_bp_ describes the dorsoventral location of the basepoints with respect to the x-y horizontal plane. A positive value of φ_bp_ indicates that the basepoint location is dorsal to this horizontal plane, while a negative coordinate indicates a basepoint location is ventral to this horizontal plane. The third basepoint coordinate is the radius (r_bp_). The variable r_bp_ is defined as the straight-line distance between the origin (the intersection of the above-described horizontal and coronal planes with the mid-sagittal plane) and the basepoint. The basepoint radii define the size of the rat’s mystacial pads.

#### Quantifying 3D whisker angles of emergence

The angles that describe the orientation of the whiskers as they emerge from the mystacial pad at their basepoint locations are called the angles of emergence. In previous studies, the angles of emergence were defined in terms of projection angles, and the corresponding Euler angles were provided in Supplementary Information [33]. In the present work, we chose to describe the whiskers using Euler angles applicable to the right array.

The angle θ_w_ describes the rostrocaudal orientation of the proximal, approximately linear portion of the whisker. An angle of θ_w_ = 180° points in the rostral direction, parallel to the positive y-axis, while an angle of θ_w_ = 0° points caudally, parallel to the negative y-axis (Fig 2E). The angle φ_w_ describes the elevation of the proximal, approximately linear portion of the whisker. A φ_w_ angle of 90° points dorsally, parallel to the positive z-axis, while an angle of -90° points ventrally, parallel to the negative z-axis (Fig 2F). Because whiskers have intrinsic curvature, they require a third angle, ζ_w_, to describe the roll about their own axis. An angle of ζ_w_ = 180° orients the whisker concave upward, ζ_w_ = 90° is concave forward, 0° downward, and -90° backward (Fig 2G). Note that the orientation of the whisker’s curvature in the laboratory frame depends on all three orientation angles, not just ζ_w_.

We performed an optimization using the built-in Matlab^™^ function “fmincon” to determine the angles of emergence. The variables θ_w_, φ_w_, ζ_w_, along with S and A, were chosen as the optimization parameters. Constraints for the optimization were: θ_w_ = [0°, 360°], φ_w_ = [-90°, 90°], ζ_w_ = [-180°, 180°], S = [straight distance from base to tip, lower bound + 50 mm], and A = [0 to 1]. Starting with the whisker point cloud in standard position and orientation (Fig 2A, 2B and 2C), the individual points within the cloud were sorted by distance from the basepoint, and their x, y, z coordinates were smoothed with a moving average filter (window size of five samples). The whisker was then resampled into 500 micron segments and translated to the origin. Next, the routine varied five parameters, θ_w_, φ_w_, ζ_w_, S, and A to fit an idealized whisker model to the whisker point cloud. The idealized model began with the base at the origin, and the proximal portion of the whisker aligned along the negative y-axis. The model’s initial orientation was concave down. To match the idealized model with the whisker point cloud, we performed a rotation sequence. The order of the Euler rotations was y-axis (roll, ζ_w_), x-axis (pitch, φ_w_), z-axis (yaw, θ_w_). All rotations were extrinsic about the global y-x-z axes. Additionally, the arc-length and curvature were varied to best match the idealized model to the point cloud. The arc-length and curvature parameters described in the 3D optimization were used only as a means to improve the whisker fitting and were not used for analytical purposes. All S and A results were derived from the 2D whisker traces. The optimization’s cost function minimized the mean sum squared distance between the whisker point cloud and the points in the idealized whisker model.

### Statistical analysis: Developing equations for morphological parameters

#### Arc length and intrinsic curvature as functions of θ_bp_ and φ_bp_

A total of 158 measurements of arc length were obtained from the 2D traces in Dataset 1 and 226 measurements from the 2D traces in Dataset 2, for a total of 384 unique observations of arc length. To identify outliers, the whiskers were grouped by row and column identity, and the mean and standard deviation for each group were calculated. If an individual whisker was greater than two standard deviations above or below the mean, that whisker was eliminated from analysis. Out of 384 whiskers, 27 outliers (5.72%) for arc length were removed, yielding a total of 357 whiskers available for further analysis.

A total of 130 measurements of intrinsic curvature were obtained from Dataset 1 and 222 measurements from Dataset 2, for a total of 352 unique observations of intrinsic curvature. After grouping by whisker row and column identity, 16 outliers (4.55%) for the intrinsic curvature coefficient (A) were eliminated leaving a total of 336 whiskers for further analysis.

We next aimed to construct models for S and A as functions of θ_bp_ and φ_bp_ without overfitting the data. To avoid overfitting, we did not fit a model directly to the entire dataset for each parameter. Instead, we first found the best fit model for each of the seven rats individually. The methods for selecting the best fit model were as follows:

First, we divided each dataset for S and A with outliers removed into seven subgroups by rat identity. For each of the seven rats, histograms of S and A were found to not be quite normally distributed. We analyzed the data both with and without applying log-transformations to the S and A coefficients to improve normality. Linear regression models were then constructed for each rat using both the original non-transformed and the log-transformed data.

Second, for both the original and log-transformed data, we tested whether S and A were univariately associated with θ_bp_ and/or φ_bp_. We tested the null hypothesis that the regression coefficients for θ_bp_ and/or φ_bp_ were equal to zero. If the p-value for the independent variable coefficients was less than or equal to 0.05, we rejected the null hypothesis and a second order model (square of θ_bp_ and/or square of φ_bp_) was fit and again the hypothesis that the regression coefficients for these higher order terms were equal to zero was tested. For each quadratic order coefficient in the model, if the p-value was greater than 0.05, the model remained first order. If the p-value was less than 0.05, a third order model (cube of θ_bp_ and/or cube of φ_bp_) was fit and tested for significance. If the p-value for each cubic coefficient was greater than 0.05, the model reverted to second order, but if the p-value was less than 0.05, the model remained third order. We did not find any models for whiskers from an individual rat to have independent variable predictors greater than second-order.

Third, if both basepoint parameters (θ_bp_ and φ_bp_) were univariately associated to either first or second order with either the original data or log-transformed A or S, both parameters were then included in a multivariable linear regression model for each individual rat. The method of “forward selection” was used to introduce the terms as first linear predictors. Again, the hypothesis that the regression coefficients for θ_bp_ or φ_bp_ were equal to zero was tested. If the p-value for the independent variable coefficients was less than or equal to 0.05, a second order model (square of θ_bp_ and/or square of φ_bp_) was fit and again the hypothesis that the regression coefficients for these higher order terms were equal to zero was tested. We used Akaike Information Criterion (AIC) as an additional metric to avoid overfitting. If both the independent variable coefficients were significant and the AIC for the higher order model was more than two points lower than the lower order model, the higher order model was selected.

The best fit univariate or multivariable linear regression model was compared across all seven rats for the original data and log-transformed A or S. An overall model, based on data from all rats together, was fit only after taking into account the results of the models fit to each individual rat. This model was fit similarly to the individual models, except that a parameter was included in the final combined model only if it had first appeared as a significant predictor in at least six of the seven individual rat models. If a parameter met this criterion it was initially included in the model and retained or discarded using the same forward selection procedure described for the individual models. The order of the combined model was not allowed to exceed the highest order of the six out of seven individual rat models to avoid overfitting.

#### Euler angles of emergence (θ_w_, φ_w_, and ζ_w_) and r_bp_ as functions of θ_bp_ and φ_bp_

Together, Datasets 1 and 2 contained a total of 518 whiskers, but 3D data was not obtained for two whiskers in Dataset 2. Thus, a total of 516 3D basepoint coordinates (r_bp_ θ_bp_ φ_bp_) and 3D angles of emergence (θ_w_, φ_w_, ζ_w_), were obtained from the nine rats of Datasets 1 and 2.

To identify outliers for each parameter, the whiskers were grouped by row and column identity, and the mean and standard deviation for each group were calculated. As before, if a parameter from an individual whisker was greater than two standard deviations above or below the mean, the value of that parameter for that whisker was eliminated from analysis.

Out of 516 whiskers, 41 outliers (7.95%) were removed for r_bp_, 24 outliers (4.65%) were removed for θ_bp_, 24 outliers (4.65%) were removed for φ_bp_, 23 outliers (4.46%) were removed for θ_w_, 23 outliers (4.46%) were removed for φ_w_, and 29 outliers (6.01%) were removed for ζ_w_. Thus, after outlier removal, 475 values remained for r_bp_, 492 values remained for θ_bp_ and φ_bp_, 493 values remained for θ_w_ and φ_w_, and 487 values remained for ζ_w_. Note that each parameter was considered independently for outlier removal.

We next aimed to construct models for r_bp_ θ_w_, φ_w_, and ζ_w_ as functions of θ_bp_ and φ_bp_ without overfitting the data. To avoid overfitting, we did not fit a model directly to the entire dataset for each parameter. Instead, we first found the best fit model for the parameters r_bp_, θ_w_, φ_w_, and ζ_w_ for each of the nine rats individually. The method for selecting the best fit model was identical to the procedure for selecting the best fit model for S and A, as follows:

We first divided each dataset for r_bp_, θ_w_, φ_w_, and ζ_w_ with outliers removed into nine subgroups by rat identity. For all of the nine rats, histograms of r_bp_, θ_w_, φ_w_, and ζ_w_ were found to be normally distributed, and thus a log-transformation to achieve normality was not necessary. Linear regression models were then constructed for each rat.

Second, we tested whether r_bp_, θ_w_, φ_w_, and ζ_w_ were univariately associated with θ_bp_ and/or φ_bp_ using the forward selection procedure described previously. We did not find any models for whiskers from an individual rat to have statistically significant independent variable predictors greater than second-order.

Third, if both basepoint parameters (θ_bp_ and φ_bp_) were univariately associated to either first or second order with r_bp_, θ_w_, φ_w_, and ζ_w_, then both parameters were included in a multivariable linear regression model for each individual rat. The method of “forward selection” was used to introduce the terms as first linear predictors. Again, the hypothesis that the regression coefficients for θ_bp_ and/or φ_bp_ were equal to zero was tested. If the p-value for the independent variable coefficients was less than or equal to 0.05, a second order model (square of θ_bp_ and/or square of φ_bp_) was fit and again the hypothesis that the regression coefficients for these higher order terms is equal to zero was tested. We also used Akaike Information Criterion (AIC) as a metric to avoid overfitting. If both the independent variable coefficients were significant and the AIC for the higher order model was more than two points lower than the lower order model, the higher order model was selected.

The best fit univariate or multivariable linear regression model was compared across all nine rats for r_bp_, θ_w_, φ_w_, and ζ_w_ as a function of θ_bp_ and/or φ_bp_. An overall model, based on data from all rats together, was fit only after taking into account the results of the models fit to each individual rat. This model was fit similarly to the individual models, except that a parameter was included in the final combined model only if it had first appeared as a significant predictor in at least seven of the nine individual rat models. If a parameter met this criterion, it was initially included in the model and retained or discarded using the same forward selection procedure described for the individual models. The order of the combined model was not to exceed the highest order of the seven out of nine individual rat models to avoid overfitting.

### Quantifying facial and skull features on the rat

#### Positioning facial and skull features in standard orientation

The (x, y, z) coordinates of the facial and skull features collected using the Microscribe^™^ were imported into Matlab^™^. These coordinates were translated and rotated to match the axis conventions shown in Fig 2C.

#### Defining the lambda bregma plane

Lambda is defined by the intersection of the sagittal and lambdoid skull sutures, while bregma is located at the intersection of the sagittal and coronal sutures. In the rat, the lambdoid suture exhibits a characteristic rostral deviation from the coronal plane as it intersects the sagittal suture, thus forming a rostral-pointing triangle with an open caudal side. We recorded the three points of this triangle for lambda, and the single intersection point for bregma. The centroid value for lambda was calculated, as was the distance between the 3D point for bregma and the centroid value of lambda. The angular offset between the lambda-bregma line and the average row plane was found by taking the difference in z-coordinates between lambda and bregma and then dividing this difference by the 3D distance between bregma and lambda. The inverse sine of this ratio yields the angular offset between the lambda-bregma line and the average row plane.

#### Digitization of lateral semicircular canal orientation

The data used for the lateral canal coordinates came from serial CT scans of *Rattus norvegicus* skull, specimen M-2272, available through the digital morphology database DigiMorph.org [43]. The specimen was originally scanned along the coronal axis, for a total of 1571 slices. Each 1024 x 1024 pixel slice is 0.02961 mm thick, with an interslice spacing 0.02961 mm and a field of reconstruction of 28 mm, resulting in a resolution of 0.02734 mm in x and y (within each coronal plane slice) and interslice resolution of 0.02961 mm in z.

We recorded 3D coordinates of the bony labyrinth and other skull features in serial coronal CT images using Reconstruct^™^. Structures traced included the entire right and left bony labyrinths, the left and right external auditory meatuses, the coronal, sagittal, and lambdoid sutures, and the lateral corners of the right and left upper incisors. These data were imported into Matlab^™^. The (x, y, z) coordinates of the following key features of skull anatomy were then manually obtained: (1) lambda and bregma; (2) the locations at which each of the two lateral canals terminated in a crista; (3) the lateral-most point of each of the two lateral canals; (4) five distinct points around the circumference of each meatus; (5) the lateral corners of the incisors.

We brought the points from the CT scan data into the same reference frame as the Microscribe^™^ data by aligning a subset of corresponding points between the two datasets. These points included lambda, bregma, and the corners of the incisors. We found the rotations and translations that brought these points into register, and then applied these same rotations and translations to the points for the other features, thus bringing the points describing the bony labyrinth and external auditory meatus into the shared reference frame as well.

A plane was fit to the lateral semicircular canals bilaterally, using the Matlab^™^ function “affine_fit” [44]. This function finds the plane of best fit to a set of points based on the least squares of the normal distance of the set of points to the plane. The angle between the horizontal canal plane and the average row plane was found as the arccosine of the dot product of the normal vectors of the two planes.

## Results

As described in the introduction, a primary goal of the present work was to develop a model of the rat facial features, with special emphasis on the vibrissal array, that permitted quantitative comparisons with the arrays of other animal species. However, species differ in numbers of rows and columns of whiskers, and the whiskers span different regions of the face. It is also unclear which whiskers best correspond between species. Therefore, instead of identifying whiskers by row and column position, we aimed to identify whiskers by their three-dimensional (3D) geometric location on the animal’s face, within a chosen coordinate system. To achieve this goal required three steps, described in the first three sections of *Results*.

First, we had to “renumber” the columns of rat whiskers to ensure consistency with the numbering schemes used for other species. Second, we chose an origin and a horizontal plane, thereby establishing axis conventions by which to localize the whiskers on the animal’s face. Third, we could then assign a 3D coordinate (r, θ, φ) to all measured points on the rat’s face, including all whisker basepoints (r_bp_, θ_bp_, φ_bp_), where the subscript bp stands for basepoint.

These three steps establish r_bp_, θ_bp_, and φ_bp_ as fundamental parameters that can then be used to quantify the remaining five parameters that describe whisker geometry. Accordingly, the fourth section of results quantifies whisker arc length (S) and whisker intrinsic curvature (A) as functions of the whisker basepoints, and the fifth section of results quantifies the three angles at which each whisker emerges from the mystacial pad. These three “angles of emergence” are denoted as θ_w_, φ_w_, ζ_w_, where the subscript “w” stands for whisker.

Finally, the sixth and last section of *Results* characterizes the location of a variety of skull and facial features (e.g., lambda, bregma, eyes, nostrils, mouth, incisors, pinnae) relative to the position and orientation of the whisker array.

### Numbering the whisker columns of the rat

**Fig 3:**
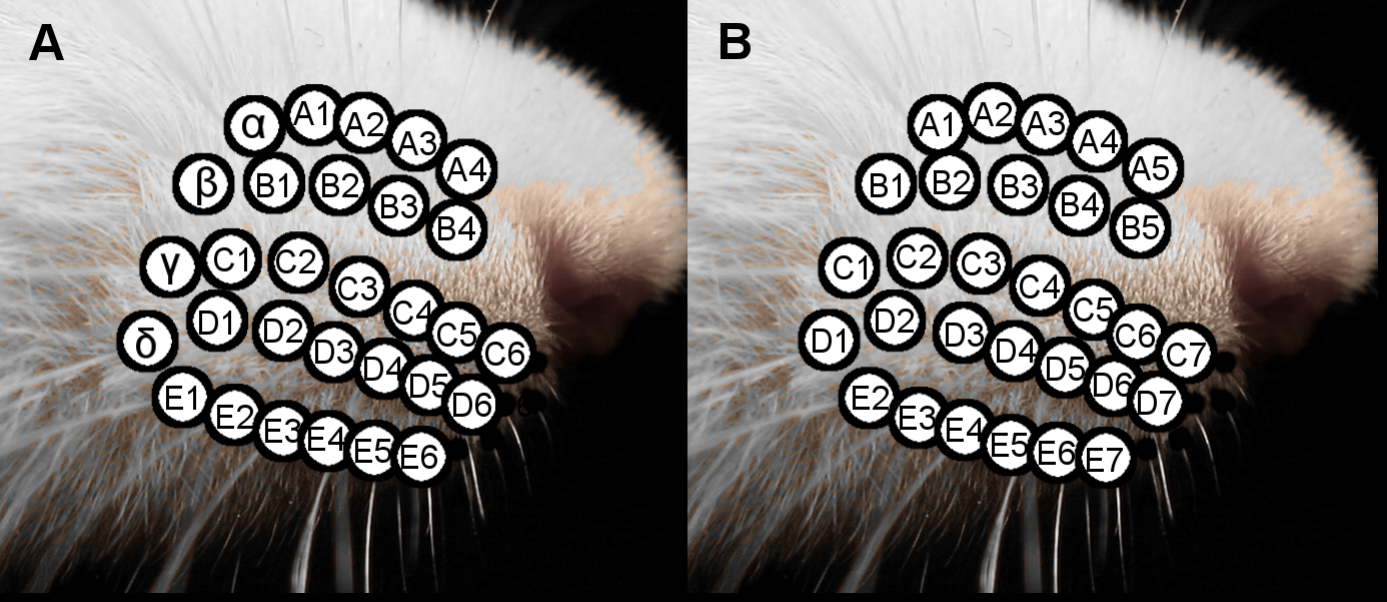
Standardized whisker nomenclature to enable cross-species comparisons. (A) Close-up of the whisker basepoints on the mystacial pad, showing the traditional nomenclature. Greek letters are assigned to the whiskers of the caudal-most arc and more rostral arcs are assigned the numbers 1–6. (B) Close-up of the whiskers of the mystacial pad, showing a nomenclature more suited for cross-species comparisons, with columns assigned values from 1–7.

The rat vibrissal array is illustrated in Fig 3A, showing the standard nomenclature. This nomenclature labels the caudal most arc of whiskers the “Greek” or “straddler” arc, and subsequent arcs are numbered from 1–6 [45–47].

However, not all species have vibrissal arrays that contain an easily identifiable Greek arc [26]. For example, the whisker array of the harbor seal has a diamond-like shape, and the caudal-most column is numbered beginning with 1 [48]. We therefore re-assigned the caudal most Greek arc of the rat a column identity of 1, with increasingly rostral columns numbered from 2–7 (Fig 3B).

### Defining 3D axis conventions: Choosing the origin and the horizontal plane

In principle, our methodology allows the origin to be placed at any location. The origin determines the location relative to which every structure is referenced, so its location should be meaningful. Two earlier studies chose the origin near the center of the nose [32–33]. However, because the current work focuses particularly on the whiskers, we chose the origin as the average of all right and left whisker basepoints. This choice specifically emphasizes distances from the center of the whisker array.

**Fig 4:**
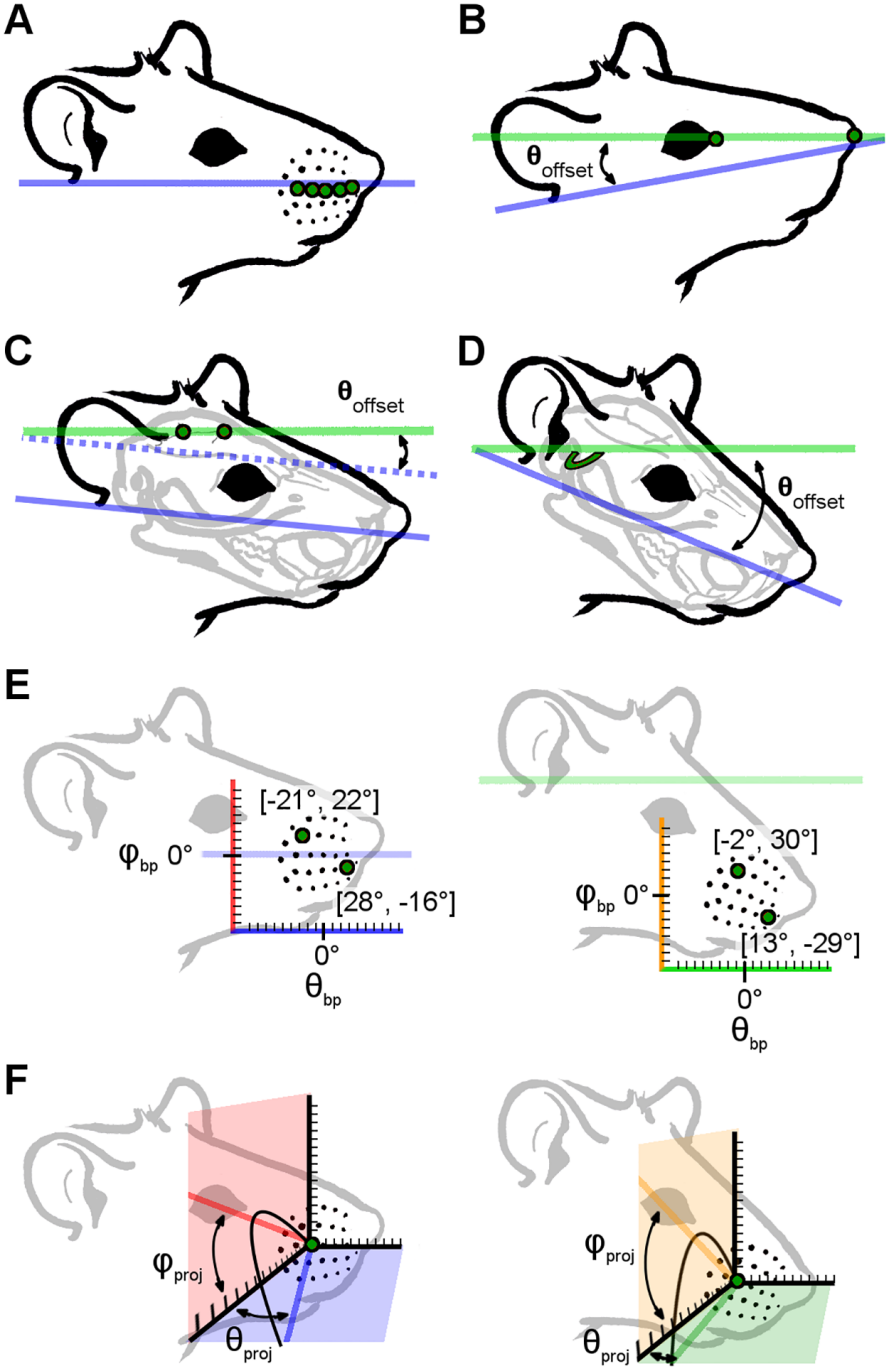
Schematics illustrating four possible choices of horizontal plane and the consequences of varying head pitch on the mathematical description of basepoint coordinates and whisker orientation. (A) The average whisker row plane is found by averaging the planes of best fit for each individual whisker row across left and right sides of the face. This plane is defined as “horizontal” in the present work. (B) Connecting the eye corners and nose yields an ~+12° offset from the average whisker row plane, tilting the rat head slightly upward. (C) The bregma-lambda plane is offset from the average whisker row plane by ~-8°, pitching the rat head slightly downward. (D) The semi-circular canal plane is offset by ~-38.5°, tilting the rat head substantially downward. (E) The left panel shows the angular basepoint coordinates (θ_bp_, φ_bp_) of two example whiskers (B2, D6) when the average row plane is defined as the horizontal x-y plane. The coordinate for B2 is (-21.5°, 22.3°) and the coordinate for D6 is (27.6°, -15.9°). The pale purple horizontal line at φ_bp_ = 0° represents the average row plane. The x-axis is also colored purple to highlight that it is parallel with the average row plane. The right panel shows the angular coordinates (θ_bp_, φ_bp_) of the same two whiskers (B2, D6) when the semi-circular canal plane is defined as horizontal. The coordinate for the B2 whisker is now (-2.2°, 30.2°) and the D6 whisker coordinate is now (13.1°, -29.5°). The pale green horizontal line indicates the semi-circular canal plane. The x-axis is also shown in green to highlight that it is now parallel to the semi-circular canal plane. Values in image have been truncated for visual clarity. (F) The left panel shows the angles of emergence for the C3 whisker, projected into the x-y plane (blue) and the x-z plane (red). These projection angles are denoted as θ_proj_ and φ_proj_. In this panel, the average whisker row plane is defined as the horizontal plane, and the blue and red vectors represent projections of the proximal (approximately linear) portion of the whisker. The right panel illustrates the same angles of emergence when the semi-circular canal plane is defined as the horizontal plane. The redefined x-y plane is shown in green and the x-z plane in orange. New projection angles for the proximal, approximately linear portion of the whisker, are illustrated by the green and orange vectors. Again, although the relative orientation of the whisker with respect to all other facial features remains constant, the projection angles describing the orientation of that whisker are affected by choice of head pitch.

The choice of horizontal plane (i.e., the plane of zero head pitch) is a similarly important yet flexible parameter. We considered four possible choices for the horizontal plane: *1)* the average row plane (the plane of actuation of the whiskers); *2)* the plane defined by the corners of the eyes and nose (a plane easily observable in behavioral studies); *3)* the plane defined by the skull landmarks lambda and bregma (a plane relevant to electrophysiological studies); *4)* the plane defined by the lateral semicircular canals (a plane relevant to cross-modal sensory processing). Each of these choices for the horizontal plane will have its own costs and benefits, and each will result in different equations relating whisker basepoints to the angles at which the whiskers emerge from the face. Ultimately, we chose the horizontal plane to be the average whisker row plane (Fig 4A) because of its clear biomechanical relevance during whisker actuation.

In considering the remaining three planes, we define a negative head pitch (snout pointing down relative to the average row plane) as a negative angle, and a positive head pitch (snout pointing up relative to the average row plane) as a positive angle.

*Connection of eye corners and nose*: An earlier study [32] defined the horizontal plane by connecting the anterior-most corners of the eyes with the top-most, anterior-most point located on the hemispheric midline of the nose (Fig 4B). For the five rats for which 3D coordinates of the eyes and nostrils were acquired, the angular offsets relative to the average whisker row plane were found to be +9.35°, +11.4°, +15.0°, +11.9°, +13.9°, yielding an average of +12.3°, a median of +11.9°, and a standard deviation of 2.20°. Selecting this orientation would on average tilt the snout up by ~12° relative to the average whisker row plane head orientation. This orientation is most similar to that used in an analysis of binocular vision of the rat [49–50].

*The bregma-lambda line*: Lambda and bregma are skull coordinates defined by the coronal, sagittal, and lambdoid skull sutures, and in the rat, they lie relatively flat on top of the skull. As illustrated in Fig 4C, defining the lambda-bregma line as horizontal would tip the rat’s head down relative to the average row plane. For the four rats for which 3D coordinates for bregma and lambda were acquired, the angular offsets were -8.51°, -9.92°, -8.61°, and -5.14°, yielding a mean of -8.04°, a median of -8.56°, and a standard deviation of 2.04°. This orientation would, on average, pitch the head down by ~8°. Although the value for the last rat appears to be relatively low, all four values are within the error range for single point measurements.

*Semi-circular canals*: As described in *Methods*, we measured the points at which the horizontal semicircular canals terminate in ampulae, yielding four points in total, two on either side of the head. The angular offset of the plane fit to these points (the semi-circular canal plane) to the average whisker row plane is approximately -38.5° (Fig 4D). Selecting this orientation would on average tilt the head down by about 38.5° relative to the average whisker row plane.

Each of these choices for the horizontal plane will have its own costs and benefits, and each will result in different equations relating whisker basepoints to the angles of emergence. A close examination of these different equations is outside the scope of the present study, but a few examples of differences in whisker basepoint coordinates and emergence angles are illustrated in Fig 4E and 4F. The left panel of Fig 4E shows basepoint coordinates of two example whiskers in standard head pitch, while the right panel shows coordinates of those same whisker basepoints after the head pitch has been changed to align the lateral semicircular canal plane with the horizontal plane. Although the relative position of all basepoints of course remains constant, the coordinates describing those points change due to the change in reference frame. The left panel of Fig 4F shows the projection of the proximal, linear portion of the whisker into the x-y plane (defining the azimuthal angle θ_proj_) and into the x-z plane (defining the elevation angle φ_proj_). As shown in the right panel, the values of these angles change with head pitch, although of course the geometry of the whiskers themselves do not change.

### Three dimensional coordinates of the whisker basepoints as functions of row and column identity

With axis conventions established, every point on the rat’s face and whiskers can be assigned a 3D coordinate. Specifically, each basepoint is assigned a coordinate (r_bp_, θ_bp_, φ_bp_). Average experimental values for these coordinates by whisker identity are provided in S1 Table. Given that the vast majority of work in this model species has been done using row and column identity [31–33], we began by quantifying the relationships between θ_bp_ and φ_bp_ and whisker row and column identity.

The azimuthal coordinate, θ_bp_, was found to be linearly related to column identity (Eq 1) and the elevation coordinate, φ_bp_, was quadratically related with row identity (Eq 2):

$$\theta_{bp}=13.4\;Col-48.6,Adj.R^{2}=0.87$$ (1)

$$\varphi_{bp}=1.49\;{Row}^{2}-26.3\;Row+68.0,Adj.R^{2}=0.95$$ (2)

In Eqs 1 and 2, Col varies from 1 to 7, Row varies from 1 to 5, and θ_bp_ and φ_bp_ are in degrees.

It is important to note that the form of these equations depends critically on the choice of origin and horizontal plane, an idea elaborated further in the *Discussion*.

**Fig 5:**
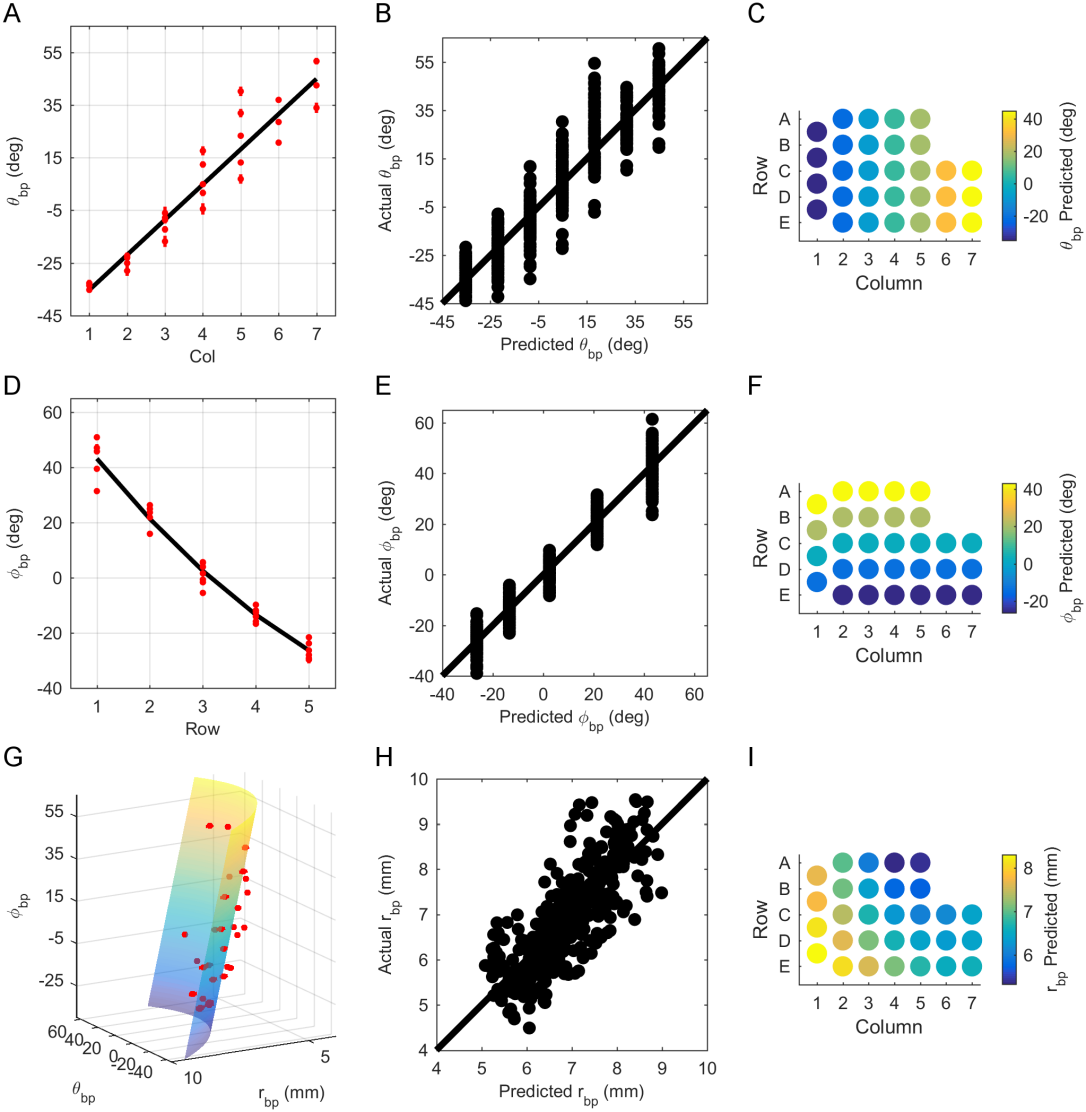
Relationship between basepoint parameters and row and column position on the array. (A) θ_bp_ increases linearly with column identity (Col). The black line represents Eq 1. Red dots show average θ_bp_ when grouped by whisker identity, with the red bars representing standard error (SE). (B) Relatively uniform dispersion of actual vs. predicted values for θ_bp_ about the identity line indicates correct model choice for Eq 1. (C) Predicted variation of θ_bp_ by column (Eq 1) when grouped by whisker identity. (D) φ_bp_ decreases as Row increases. Eq 2 is shown as a black line. Red dots represent average φ_bp_ when grouped by whisker identity, where the red bars show SE. (E) Relatively uniform dispersion of actual vs. predicted values for φ_bp_ about the identity line indicates correct model choice for Eq 2. (F) Predicted variation of φ_bp_ with Row (Eq 2) when grouped by whisker identity. (G) r_bp_ decreases with both θ_bp_ and φ_bp_. Eq 3 is shown as a 3D surface. Plotting r_bp_ on the y-axis and φ_bp_ on the z-axis demonstrates the approximate shape of the rat’s cheek. Red dots represent mean r_bp_ when grouped by whisker, and red bars show SE. (H) Relatively uniform dispersion of actual vs. predicted values for r_bp_ about the identity line indicates correct model choice for Eq 3. (I) Predicted variation of r_bp_ with row and column (Eq 3) when grouped by whisker identity.

The positive coefficient for column identity (Col) in Eq 1 indicates that θ_bp_ increases from caudal to rostral. This relationship is highlighted when θ_bp_ is plotted as a function of column (Fig 5A). Relatively uniform dispersion of actual vs. predicted values about the identity line (Fig 5B) indicates that the correct model was chosen (Fig 5B). Fig 5C shows the strong dependence on column for θ_bp_, predicted by Eq 1, by whisker identity across the array.

Eq 2 indicates that φ_bp_ decreases from dorsal to ventral, a relationship easily observed in Fig 5D, which plots φ_bp_ as a function of row. For all nine rats, a quadratic relationship between φ_bp_ and row was found to be statistically better than linear on the basis of AIC; however, it is critical to note that this relationship will change depending on the definition of the horizontal plane. Fig 5E shows relatively uniform dispersion of actual vs. predicted values about the identity line, reflecting correct model choice. Finally, the quadratic variation of Eq 2 for φ_bp_ across the whisker array is shown in Fig 5F as a colormap.

We next examined the relationship between r_bp_ and the basepoint parameters θ_bp_ and φ_bp_. Fig 5G shows r_bp_ to be quadratically related to θ_bp_ and linearly related to φ_bp_:

$$r_{bp}=0.000511\;{\theta_{bp}}^{2}-0.0295\;\theta_{bp}-0.0162\;\varphi_{bp}+6.50,Adj.R^{2}=0.65$$ (3)

where θ_bp_ and φ_bp_ are in degrees and r_bp_ is in millimeters. Although rat whisker pads curve in both the rostral-caudal and dorsal-ventral directions, qualitative observation suggests that the curvature in the rostrocaudal direction is much steeper than the curvature in the dorsoventral direction. Consistent with this appearance, we found that the equation for r_bp_ is statistically significant only to first-order in φ_bp_.

Fig 5H shows relatively uniform dispersion of actual vs. predicted values about the identity line. Dispersion was smaller than for all other fits tested, reflecting correct model choice. However, there is also high variability, as indicated by the lower adjusted R^2^ value (0.65). This variability may partly arise from size differences between rats. Fig 5I shows the predicted variation in r_bp_ when grouped by whisker identity. Note that the caudal-most and ventral-most basepoints are furthest from the origin. This occurs because the rostral region contains a denser grouping of whiskers. Each whisker basepoint is weighted identically when defining the origin as the mean position of all basepoints. The origin is therefore “pulled” slightly closer to the rostral region.

### Two-dimensional whisker shape: Arc length and intrinsic curvature coefficient

We next quantified the parameters that describe 2D whisker geometry, arc length and curvature, as functions of basepoint coordinates θ_bp_ and φ_bp_. The parameter r_bp_ was omitted because it depends more on head size than relative position of the whisker in the array. Arc length was found to be best described as an exponential function of θ_bp_:

$$S=e^{-0.0246\theta_{bp}+3.12},Adj.R^{2}=0.85$$ (4a)

Or alternatively

$$S=\frac{22.6}{e^{0.0246\theta_{bp}}},Adj.R^{2}=0.85$$ (4b)

where θ_bp_ is in degrees and S is in mm.

**Fig 6:**
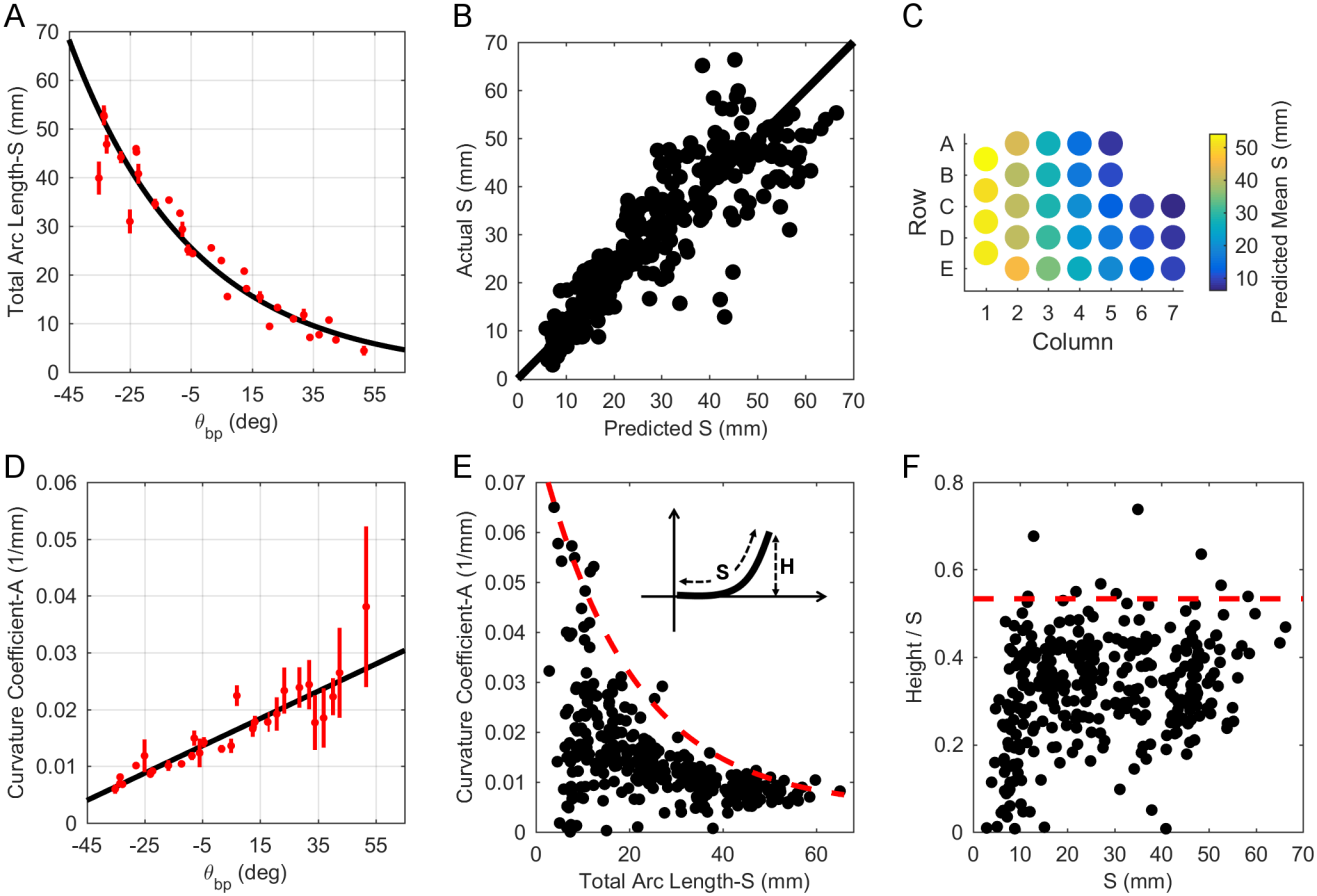
Relationship between 2D whisker geometry and basepoint parameters. (A) Whisker arc length (S) can be described as a decaying exponential function of θ_bp_, decreasing from caudal to rostral. The black line represents Eq 4a. Mean ± standard error (SE) by whisker identity is shown in red. (B) Relatively uniform dispersion of actual vs. predicted values for S about the identity line indicates correct model choice for Eq 4a. (C) When grouped by whisker identity, Eq 4a predicts that arc length decreases with column position. (D) Intrinsic curvature coefficient (A) can be described as a linearly increasing function of θ_bp_ from caudal to rostral. The black line represents Eq 5. Mean ± SE by whisker identity is shown in red. (E) Plotting A vs. S highlights that shorter whiskers have higher variability in curvature. This relationship is bound by the curve given by Eq 6. Inset: The upper bound on A constrains the “height” (H) of a whisker. (F) The height (H) of the whisker tip does not typically exceed more than 53.3% of the whisker’s arc length (S).

The negative coefficient for θ_bp_ in Eq 4a indicates that S exponentially decays from caudal to rostral. This relationship is shown in Fig 6A. Consistent with the intuition provided by the form of Eq 4b, the figure shows that S is ~23 mm for central whiskers, where θ_bp_ = 0, with experimental values of S varying between 2.9 and 66.3 mm over the entire pad. Relatively uniform dispersion of actual vs. predicted values for S about the identity line indicates correct model choice for Eq 4a (Fig 6B). Finally, Fig 6C provides an intuition for variations in S across the array as predicted by Eq 4a and when grouped by whisker identity. To compare with earlier studies, an expression for S as a function of column can be found in Supplementary Information.

Intrinsic whisker curvature (A) was quantified using the parameterization y = Ax^2^ (see Methods). No strong relationship between A and the basepoint coordinates was found. The best relationship described A as a linear function of θ_bp_:

$$A=0.000240\;\theta_{bp}+0.0148,Adj.R^{2}=0.34$$ (5)

where θ_bp_ is in degrees and A is in units of 1/mm. The low adjusted R^2^ indicates that the fit is poor, confirmed in Fig 6D. The figure shows that the fit is particularly poor for rostral whiskers, which exhibit much higher variability in curvature than caudal whiskers. Overall Eq 5 yields a reasonable approximation to whisker shape (S1 Fig), but the relationship is not strong.

To explore the origin of the poor fit, we investigated the relationship between curvature and arc length, as shown in Fig 6E. The relationship for A as a function of S was worse than that found for Eq 5. Instead, Fig 6E suggests that a whisker’s intrinsic curvature is not fixed by an average value, but rather forced to lie below an upper bound (c.f., [51]).

To compute this upper bound, we performed a sliding window analysis. We found the maximum value of A for arc lengths between 0–4 mm, then for arc lengths between 1–5 mm, and so on, up to the window S = 62–66 mm. This analysis yielded a curve based on the maximum value of A in each window. The best equation was fit to those maximal points and found to be:

$$A=0.0746\;e^{-0.0506S}+0.00479,Adj.R^{2}=0.95$$ (6)

In general, whisker curvature will not be greater than values that lie along this curve.

The upper bound on the value of A tightly constrains the height (H) of a whisker relative to its arc length (S) (Fig 6E, inset). This effect is quantified in Fig 6F, which shows the ratio of height to arc length, plotted as a function of arc length. In this figure, black dots represent data from all whiskers for which 2D geometry was measured, and the red dashed line indicates the upper bound on the data at a constant value of 0.533. This line was found using the same moving average window used to calculate the bound in Fig 6E, and indicates that the vertical distance of the whisker tip does not extend a height greater than 53.3% of the whisker’s arc length.

### Angles of emergence of the whiskers as a function of basepoint coordinates

The 3D angles of emergence (θ_w_, φ_w_, ζ_w_) were quantified as functions of the basepoint coordinates θ_bp_ and φ_bp_.

The azimuthal angle of emergence, θ_w_, was best described as a linear function of both θ_bp_ and φ_bp_:

$$\theta_{w}=0.598\;\theta_{bp}-0.314\;\varphi_{bp}+67.4,Adj.R^{2}=0.64$$ (7)

where θ_w_, θ_bp_, and φ_bp_ all have units of degrees.

**Fig 7:**
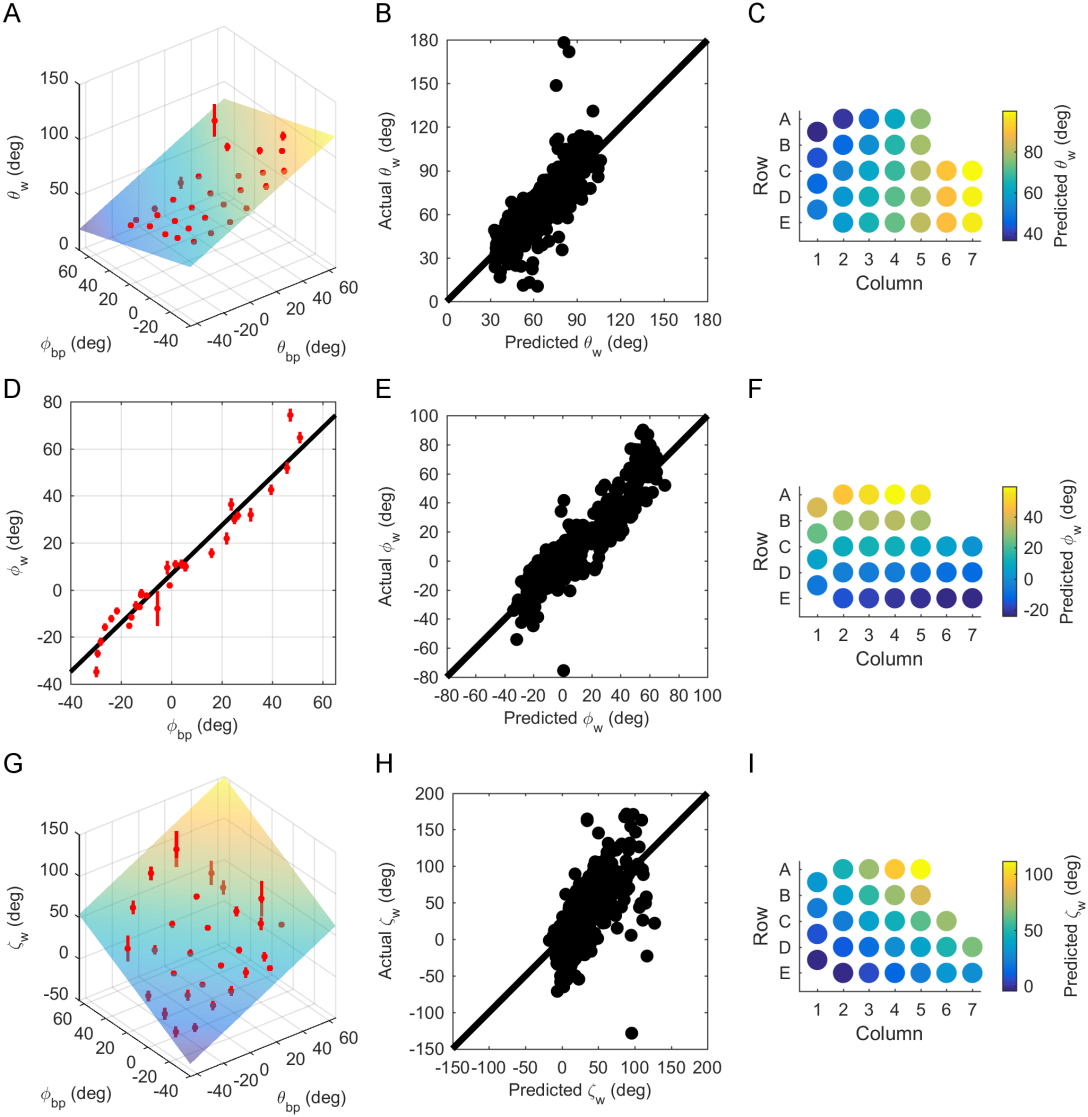
Relationship between whisker angles of emergence and basepoint parameters. (A) θ_w_ is a linear function of θ_bp_ and φ_bp_. Mean ± standard error (SE) by whisker identity is shown in red. (B) Relatively uniform dispersion of actual vs. predicted values for θ_w_ about the identity line indicates selection of the correct model choice for Eq 7. (C) Eq 7 is plotted as a colormap to show the variation of θ_w_ across the array. (D) φ_w_ can be described as a linear function of φ_bp_. Mean ± SE by whisker identity is shown in red. (E) Relatively uniform dispersion of actual vs. predicted values for φ_w_ about the identity line indicates selection of the correct model for Eq 8. (F) Eq 8 is plotted as a colormap to show the variation of φ_w_ across the array. (G) ζ_w_ can be described as a polynomial function linear in φ_bp_ and linear in θ_bp_. Mean ± SE by whisker identity is shown in red. (H) Relatively uniform dispersion of actual vs. predicted values for ζ_w_ about the identity line indicates selection of the correct model for Eq 9. (I) A colormap shows how Eq 9 varies across the array.

The positive coefficient for θ_bp_ indicates that θ_w_ increases from caudal to rostral, and the negative coefficient for φ_bp_ indicates that θ_w_ decreases from ventral to dorsal. These effects are shown in Fig 7A, and the relatively uniform dispersion of actual vs. predicted values for θ_w_ about the identity line indicates correct model choice for Eq 7 (Fig 7B). Variations in θ_w_ across the array are shown in Fig 7C. This figure confirms the intuition that whiskers in columns 1–3 are oriented more caudally than whiskers in columns 4–7, but also reveals a small but consistent effect: the whiskers are oriented slightly more caudally for the A and B rows than for the D and E rows. This effect is small but was found for all nine rats. A 5° increase in θ_bp_ (approximately the angular offset between adjacent whisker basepoints) will increase θ_w_ by ~3°, while a 5° increase in φ_bp_ will decrease θ_w_ by ~1.6°.

The elevation angle, φ_w_, was not dependent on θ_bp_, varying linearly only with φ_bp_:

$$\varphi_{w}=1.04\;\varphi_{bp}+6.68,Adj.R^{2}=0.85$$ (8)

where φ_w_ and φ_bp_ are in degrees.

The positive coefficient in front of φ_bp_ in Eq 8 indicates an increase in φ_w_ from ventral to dorsal, a relationship shown in Fig 7D. Relatively uniform dispersion of actual vs. predicted values for φ_w_ about the identity line indicates correct model choice for Eq 8 (Fig 7E), and Fig 7F shows the prediction of Eq 8 for φ_w_ when grouped by whisker identity. Interestingly, the range of φ_w_ is about the same as the range of θ_bp_. Whiskers emerge at θ_bp_ from -43.9° to 60.5° (a range of ~104°), and at φ_w_ between -39.0° and 61.4° (a range of ~100°). This result indicates that the array fans out equally in both dorsal-ventral and rostral-caudal directions.

Finally, the angle ζ_w_ was best described as a linear function of both θ_bp_ and φ_bp_:

$$\zeta_{w}=0.876\;\theta_{bp}+0.845\;\varphi_{bp}+37.9,Adj.R^{2}=0.42$$ (9)

where ζ_w_, θ_bp_, and φ_bp_ are in degrees. This relationship holds only for whiskers with arc length (S) greater than or equal to 8 mm. Whiskers shorter than 8 mm had too few data points acquired along their length to generate an accurate curve fit, and were not included in this analysis.

Eq 9 predicts ζ_w_ fairly well for whiskers more centrally located within the array. This is reflected first in Fig 7G, and also in Fig 7H, by the somewhat even dispersion of points about the identity line for predicted vs. actual ζ_w_ values. However, Eq 9 is a poor fit for whiskers further from the center of the array, and the data has high variability, as indicated by the low adjusted R^2^ (0.42). Nonetheless, Eq 9 serves as an acceptable first order prediction of the mean value of ζ_w_. The positive coefficients for θ_bp_ and φ_bp_ suggest that ζ_w_ increases from caudal to rostral and from ventral to dorsal. Fig 7I also shows this relationship; when the predicted values for ζ_w_ from Eq 9 are grouped by whisker identity, ζ_w_ is highest in rows A-C and columns 6 and 7.

The large variability in ζ_w_ is likely a result of measurement error. In contrast to θ_w_ and φ_w_, which represent rotations within a plane, ζ_w_ represents the twist of the whisker about its own axis. This angle is inherently more sensitive to small deviations in the raw data points collected using the Microscribe^™^. Although a detailed analysis lies outside the scope of the present work, we predict that the fit for ζ_w_ could improve given another choice of head pitch. This idea is elaborated further in the discussion.

### The final vibrissal array model in the context of other facial features

**Fig 8:**
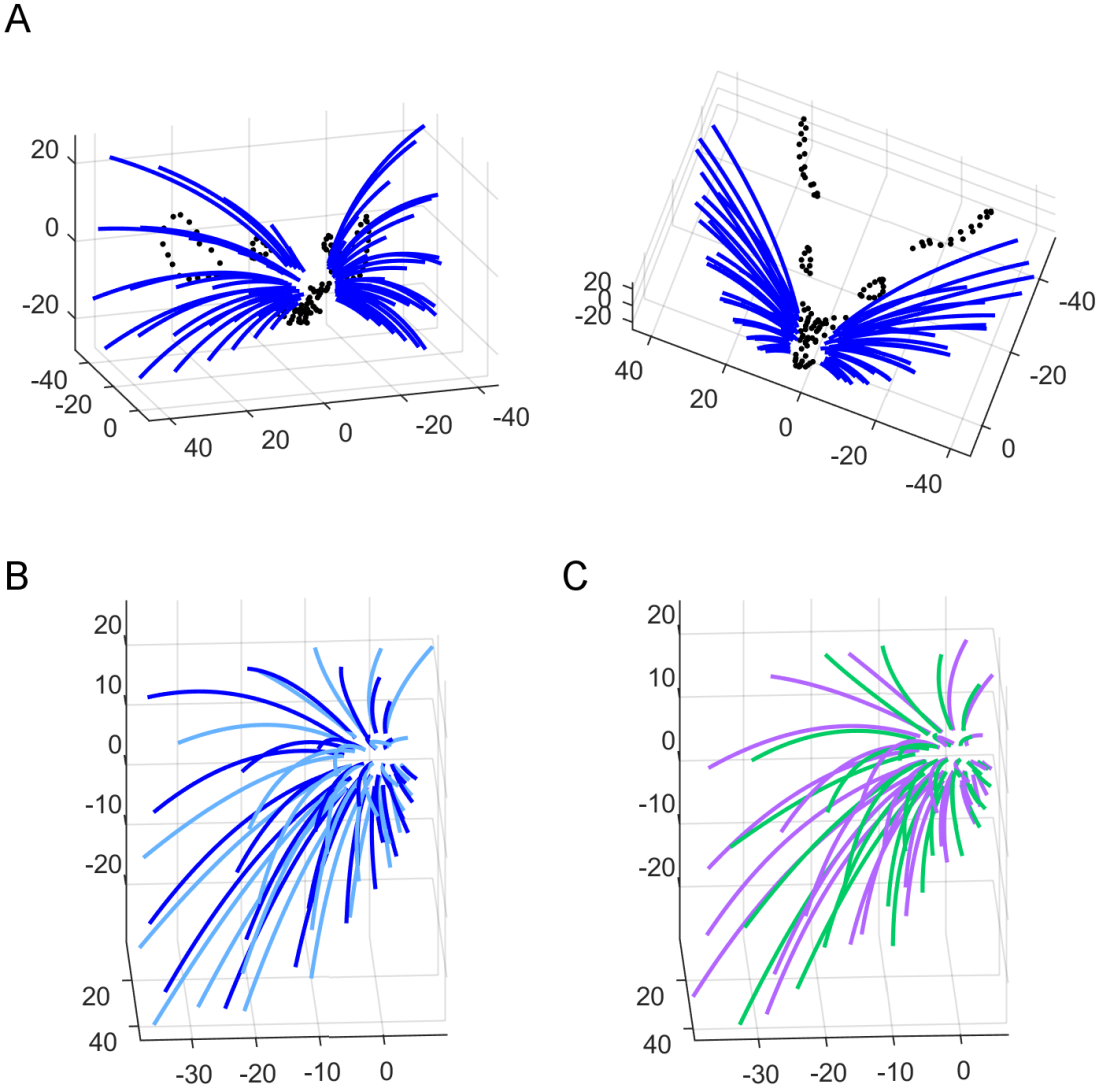
Comparison between the equation-based model and both the average and individual rats. (A) Front-on and top down views of the full whisker array model are shown in blue. A detailed trace of facial features collected from one rat (black dots) has been superimposed. (B) Equation-based model (blue) vs. smoothed traces from the averaged rat (cyan), allowing visual assessment of the model quality. (C) Smoothed traces from two individual rats superimposed, allowing visual assessment of the variability between individual animals.

Using the experimental basepoint parameters from S1 Table and Eqs 3–5 and 7–9 from *Results*, we constructed a full 3D model of the rat whisker array and superimposed the facial features obtained from one rat for visual context (Fig 8A). To provide an intuition for error in the model, Fig 8B compares the model with the “average array” found by taking the mean of individual fits to each whisker across all nine rats. This error can be visually compared with the difference between individual fit whiskers for two of the nine rats, shown in Fig 8C.

We quantified the error in Fig 8B and 8C by finding the Euclidian distances between the tips of each corresponding whisker. This error metric pools all sources of variance that affect tip position, including emergence angles, length, curvature, and basepoint location. The average rat and our model differ on average by 5.47 ± 3.28 mm (mean ± standard deviation) between corresponding whisker tips, while the two individuals differ by 4.55 ± 2.79 mm. The large standard deviation indicates that our model is as close to the average rat as two individuals are to each other.

One concern with this error metric might be that basepoint location is identical between the model and average array, but varies slightly between the two individual rats. Although this discrepancy means that the two error estimates are not strictly perfectly comparable, it is likely that the difference in basepoint location serves to increase some tip distances but decrease others, so over the total 30 whiskers the effect is small.

Both the average rat whisker array and our statistical model can be considered equally valid, though qualitatively different, ways of approximating the central tendency of the population distribution of rat whisker positions and orientations. Although the mean array might be more similar to any given individual rat from our sample, the equation-based model allows quantitative comparisons across species.

### Relationship of facial markers to basepoint parameters (θ_bp_ and φ_bp_)

Our measurement procedures for Dataset 2 included quantifying the location of facial and skull features on the array relative to the whisker basepoints. These data provide important context in which to place the whisker array and allow for cross-modal comparisons in future work.

**Fig 9:**
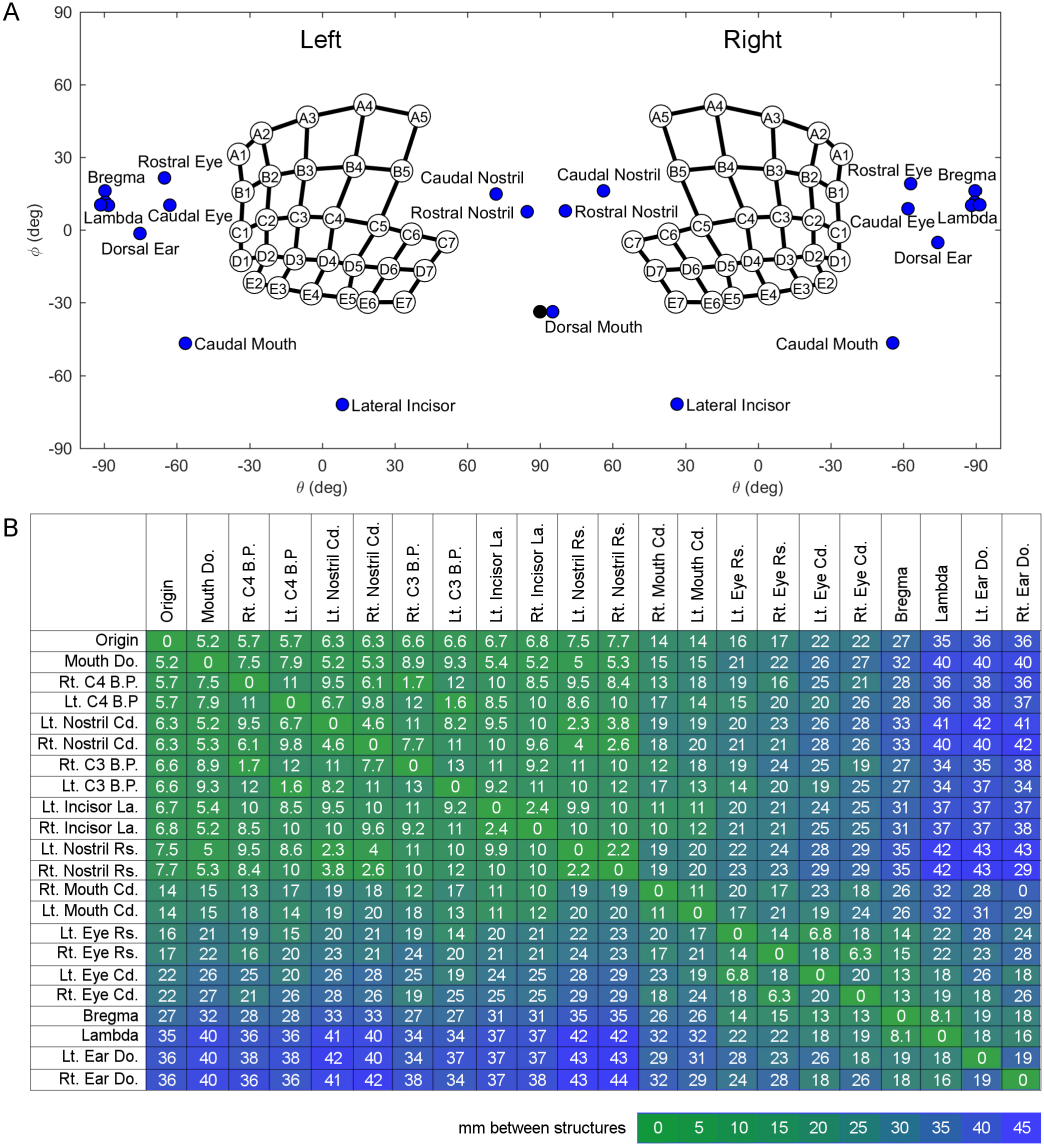
Quantification of coordinates of whisker basepoints, skull and facial features, and distances between these structures. (A) Position of the eyes, pinnae, nostrils, mouth, incisors, and bregma and lambda on the rat using coordinates θ and φ. The whisker array has been aligned into standard position and orientation using the average row plane. θ and φ are measured from the origin representing the average of all matched left and right whisker basepoints. The black dot indicates the theoretical dorsal mouth location. Notice that in this figure, the right and left facial features were not averaged. The left [right] facial features represent the average of the left [right] sides of five rats. (B) Average straight-line distances (mm) between facial features. Green indicates smaller distances, while blue indicates larger magnitudes. Entries in the array are sorted by proximity to the origin. Abbreviations: Lt. = left, Rt. = right, B.P. = basepoint, Cd. = caudal, Rs. = rostral, Do. = dorsal, La. = lateral.

Fig 9A provides the reader with an intuitive presentation of the anatomical layout of the rat’s face from the “point of view” of the whiskers. This figure shows the 2D angular locations of facial and skull features relative to the origin, i.e., the center of all whisker basepoints. To interpret this figure, you should imagine yourself as the rat, with your nose placed near the nostrils, looking into the page. Notice that the mouth spans nearly the entire horizontal axis (from θ_bp_ = -60° on the left to θ_bp_ = -60° on the right), and that the nose is central and almost completely surrounded by whiskers, while the eyes and ears are proportionately much smaller and at extreme horizontal angles. Note that the relative emphasis of these different facial features is a direct result of having chosen the origin as the center of the whisker basepoints.

Complimenting these angular distances, Fig 9B shows the 3D Euclidean distance between facial features, including two whiskers, C3 and C4 (C2 and C3 in standard nomenclature). The C4 whisker was chosen because it is closest to the origin, and the C3 whisker was chosen because it has been used in many physiological studies [39,52–55]. These distances were calculated first on each individual rat for which facial and skull feature measurements were extracted, and then each computed distance was averaged across all rats. Notice again that features of the mouth, nose, and whisker pad tend to cluster, as indicated by the block of green in the upper left, while the eyes and ears are farther away from the origin, indicated by the blue regions in the upper right and lower left. Also, the eyes and ears tend to cluster together, as indicated by the second green block at the lower right.

Some interesting comparisons can be made using Fig 9B. For example, if one compares distances between complementary left and right sensory structures, the distance can be seen to increase from nostrils (2.2 mm between right and left) to mouth (11 mm), followed by eyes (14 mm), and corners of the pinnae (19 mm). These distances are likely to reflect the importance of bilaterality for distal as opposed to proximal senses.

## Discussion

### Establishing conventions to enable cross-species comparisons of facial morphology

The specific goal of the present work was to quantify the morphology of the rat’s facial features and vibrissal array in a manner that permits cross-species comparisons. Ideally, as in the present work, comparisons of vibrissal morphology will involve quantification based on the basepoint coordinates of the whiskers; however, this level of precision may not always be possible. Some studies may require comparisons using only relative position within the array, i.e., row and column identity. Therefore, it is essential that the row and column designations given to whiskers are comparable across species.

This requirement is problematic given the nomenclature traditionally used to identify rodent vibrissae: whiskers in the caudal most arc are termed the “Greek” vibrissae (denoted α through δ), with the other vibrissae given alphanumeric labels indicating row and column identity [46]. Although this nomenclature makes good sense from a biomechanical point of view (the Greek vibrissae have intrinsic muscles that interdigitate with fibers of extrinsic muscles [56]), it is not consistent with the numbering system used for other species (e.g., the harbor seal). We therefore renumbered the whiskers within the array (Fig 3) and defined a coordinate system robust to variations in whisker geometry (Fig 2). Our decision to rename the Greek arc with the number 1 comes at the cost of acknowledging the special muscular anatomy of these whiskers, but is required for a comparative approach.

For similar reasons, the present work also establishes new axis conventions that provide an intuitive sense for the location of the mystacial pad relative to other facial features. The coordinate system shown in Fig 2 is not appropriate for mechanical simulations, but emphasizes the bilateral symmetry of the head, and ensures that corresponding right and left vibrissae have the same angular coordinates. Because different species have different numbers of rows and columns of whiskers, and some species (e.g., perissodactyls), lack a grid like arrangement entirely [20,57] the basepoint coordinates θ_bp_ and φ_bp_ are used as fundamental parameters for quantification, replacing row and column identity. For the majority of animals, which have grid-like arrays, choosing the average row plane as horizontal (Fig 4) will ensure that θ_bp_ is strongly related to column and φ_bp_ to row (Eqs 1 and 2).

### Whisker length and shape: Intrinsic curvature constrains the whisker’s “height”

Even a cursory glance at a rat’s face reveals that caudal whiskers are longer than rostral whiskers [31,33,58–59]. There is some controversy, however, as to whether the length of the whiskers more accurately follows a linear or an exponential fit as a function of basepoint coordinates or whisker identity [31,33,58–60]. The present work finds that the arc length varies as an exponential function of θ_bp_ (Eq 4), as well as column (S1 Appendix). We suggest that the origin of the controversy is that the variance in the data is just large enough to yield a high R^2^ value for either an exponential or linear fit. To determine the appropriate fit requires comparing the raw data to predicted values from both linear and exponential models to ensure selection of the correct fit based on uniform dispersion about the identity line.

The present work also provides new insights into the possible functional significance of the intrinsic curvature of the whisker. When plucked from the animal, the first 60–70% of a whisker lies in a plane and, when appropriately aligned in a standard Cartesian coordinate system, is well fit by the equation y = Ax^2^, where A is termed the intrinsic curvature coefficient. The value of A is highly variable for the shorter whiskers [32–33,51]. Although fits between A and the whisker’s arc length, row, column, or basepoint coordinates are poor, there is a strict upper bound on the value of A for a whisker of a given arc length [51, present study].

We conjectured that the upper bound on arc length (Fig 6E) might serve to constrain the “height” of the whisker, i.e., the vertical distance of the tip when the whisker is aligned with the x-axis (inset to Fig 6E). This conjecture was confirmed in Fig 6F: the ratio of height to arc length as a function of arc length is constrained to lie below 53.3%. Only 2.7% (9/332) of whiskers in the present study had a height to arc length ratio greater than this value. To summarize: the curvature coefficient for a given whisker identity can vary considerably, but must be small enough to ensure that the height of the whisker is less than ~53% of its arc length. Although the fit between A and S is poor (Eq 5), it yet yields whisker shapes that are within experimental variability for that whisker identity (S1 Fig).

These findings lead to two predictions. First, we predict that rats exhibiting a mutation such as “rex,” which leads to particularly curly whiskers [61] might be more clumsy than wild type rats. This prediction is confirmed anecdotally by breeders of “fancy rats,” which exhibit this mutation. Second, we predict that species with an ethology different from rats might have a different bound on the curvature. Accordingly, chinchillas and viscachas, both medium-sized herbivorous alpine rodents, appear to have much straighter whiskers than rats.

### Arrangement of facial features relative to the vibrissal array

The present work improves on previous models of the rat head and vibrissal array, and its deviation from the average rat is comparable to the variability observed between individual rats (Fig 8). The present results also extend previous studies to quantify the locations of different facial features relative to the vibrissal array (Fig 9). It is important to note that Fig 9 illustrates a view of the rat’s face that is a particularly “whisker-centered” in two respects. First, the angular coordinates of all facial features are plotted with respect to a whisker-centered origin, namely, the average of the whisker basepoints. Second, the average row plane was defined as horizontal (thereby defining relative values for θ_bp_ and φ_bp_) specifically because it is the primary plane of actuation for the whiskers.

**Fig 10:**
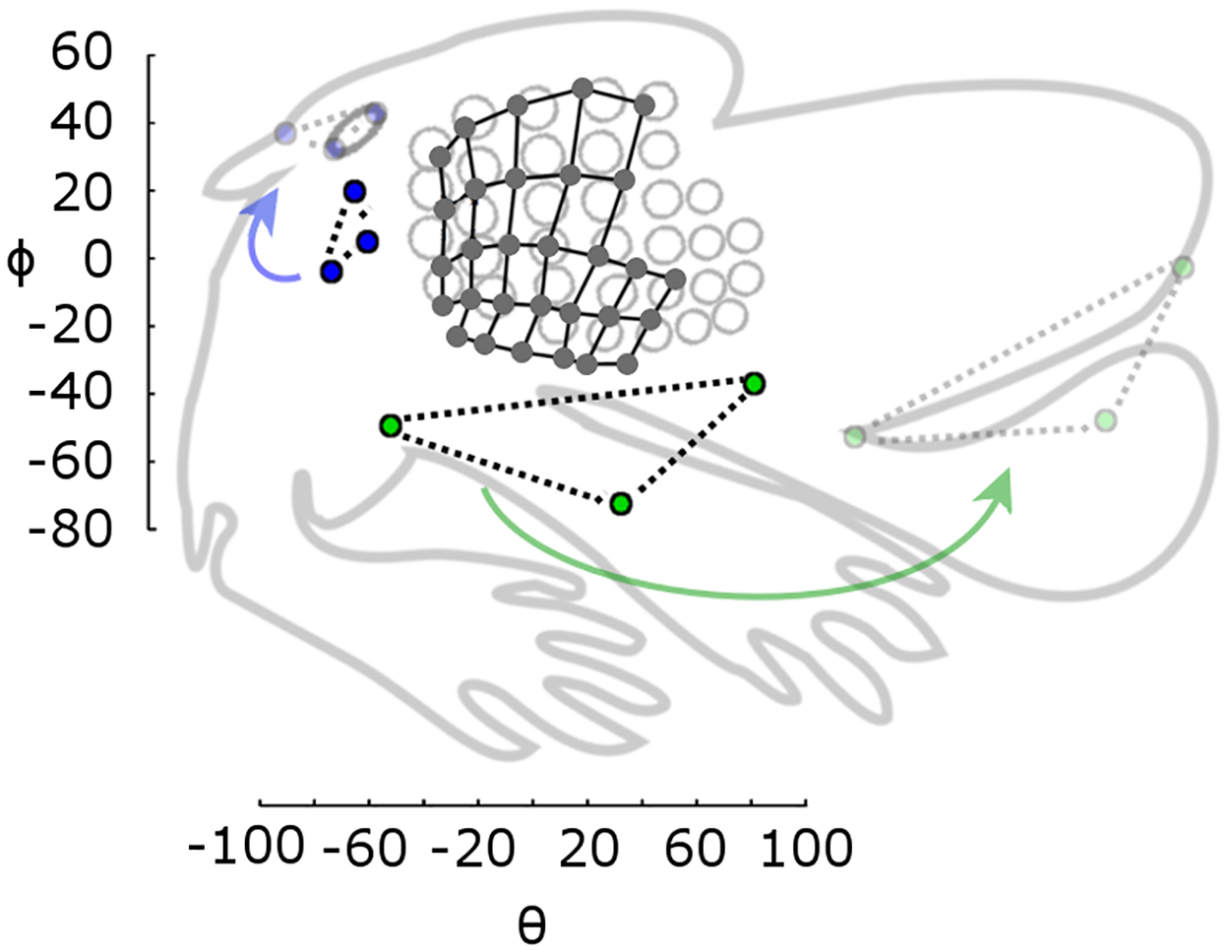
Proportion of angular area of facial features corresponds with proportion of cortical area. The ratunculus (grey outline, adapted from [62]) is rotated and scaled to approximately align the barrel representations (grey circles) with the angular locations of the basepoints from the present study (black circles connected by black grid lines). Blue points represent angular locations of the rostral and caudal points of the eye, and the dorsal corner of the pinna. When these are translated and rotated (but not scaled) they align with the features on the ratunculus (light blue circles). Similarly, the green points, representing the rostral and caudal corners of the mouth and the incisors, align with those features after repositioning (but not scaling). The nose shows a similar pattern but is not shown for visual clarity.

With this “whisker-centeredness” in mind, it is informative to compare the distances between structures with the “ratunculus” [62] that characterizes primary somatosensory cortex (Fig 10). When the whisker basepoints are aligned and scaled to match the rat barrel cortical representation, other facial features occupy proportionately about the same area in cortical real-estate as they do in angular coordinates in our model, *without* scaling. This result hints at the idea that the rat’s sensorium may be “whisker-centered.” If we had chosen the origin to be the midpoint of the eyes, for example, the angular area spanned by the mouth would not match the corresponding area in the cortical map.

### The effects of head pitch and origin

Our axis conventions place the origin at the center of the array and align the horizontal plane with the axis of actuation of most whiskers (the average row plane). With these conventions, the azimuthal angle of emergence, θ_w_, varies with both θ_bp_ and φ_bp_ (Eq 7), while the elevation angle of emergence, φ_w_, varies only with φ_bp_ (Eq 8). Both relationships are quite strong, with adjusted R^2^ of 0.643 and 0.847, respectively. This geometry means that whiskers within a single row will have different values of θ_w_, but they will have approximately the same values of φ_w_. Whiskers within a single column (arc) will contain five unique values of φ_w_, and every column will have roughly those same five φ_w_ values.

Assuming that the rat holds its head stationary, whisking will cause successive columns of whiskers to pass through roughly the same column of space. This allows the rat to sample the space with a series of whiskers that have approximately the same distribution of φ_w_ values, potentially facilitating the comparison of information gathered from different columns of whiskers. Thus, this arrangement of φ_w_ may set up each column as an easily comparable fan of sensors.

In contrast to the strong relationships found between θ_w_, φ_w_, and the whisker basepoint locations, ζ_w_ is not well predicted by either θ_bp_ or φ_bp_ (Eq 9, Adj. R^2^ = 0.42). One possible reason for this low correlation is that ζ_w_ is more sensitive to measurement error. However, we also strongly suggest that a better fit for ζ_w_ might be found for a different choice of horizontal plane. This suggestion may seem unintuitive, because the inherent geometry of an animal’s face does not change with head pitch. The key insight here, however, is that the sensing space for an animal requires not only a description of its sensory organs relative to each other, but also relative to gravity. In other words, the orientation of an animal’s sensors in world coordinates affects the sensory data that the animal acquires; thus the choice of “horizontal” is critical to morphological quantification.

In this context, it should be noted that the horizontal plane in the current work was specifically chosen so as to highlight variation with regard to the plane of vibrissal actuation in the rat. However, this plane may not be optimal for describing every aspect of the vibrissal system, different sensory modalities, or different species, especially those that do not whisk. This issue should be explored in future work.

In all, the current work describes the rat vibrissal array in detail in a way that is easily compared to other species, and establishes a foundation for future work to compare morphologies across different modalities. For example, a recent study found that the tips of the whiskers form a portion of a sphere whose center lies at the midpoint of the rat’s eyes [63]. One might ask whether a similar geometry holds for carnivores and lagomorphs, which presumably use their whiskers differently than rodents. Insights obtained through this type of comparative work could potentially contribute to bioinspired engineering solutions to problems of sensation, and will deepen our understanding of the rich diversity of animal life that surrounds us.

## Supporting information

S1 Fig Eq 5 captures approximate whisker shape, but intrinsic curvature is highly variable, even for whiskers with the same row and column identity. In all subplots, both axes have units of millimeters. Axes are square and equal so that the whisker aspect ratio is depicted accurately. Each subplot shows traces for a different whisker identity (row and column). Blue traces illustrate the scanned whiskers, smoothed and oriented to align with the x-axis as described in *Methods*. Green traces represent the fit for each whisker based on Eq 5 from the main text. To create each equation-based whisker, x values were obtained from each experimentally-measured whisker and the equation y = Ax^2^ was then plotted. There are exactly as many green traces (equation-based whiskers) in each subplot as there are blue traces (experimentally-measured whiskers). However, there appear to be fewer green traces because they overlap each other a great deal. The overlap occurs because θ_bp_ is very similar for all whiskers with a given row and column identity. This figure illustrates that Eq 5 captures the approximate shape of the whiskers, but cannot capture the high variability in whisker curvature, especially for the more rostral whiskers. Intrinsic whisker curvature is bounded by a strict upper threshold (Fig 6E), but exhibits high variability below that threshold.
(TIF)

S1 Table Average r_bp_, θ_bp_, and φ_bp_ by whisker identity (ID). (PDF)

S1 Appendix Equations as functions of row and column position. (PDF)

S1 Dataset Data to make Fig 5. (XLSX)

S2 Dataset Data to make Fig 6. (XLSX)

S3 Dataset Data to make Fig 7. (XLSX)

S4 Dataset Data to make Fig 8. (XLSX)

S5 Dataset Data to make Fig 9. (XLSX)
