# Correction: Quantifying the three-dimensional facial morphology of the laboratory rat with a focus on the vibrissae

**DOI:** 10.1371/journal.pone.0307612

**Published:** 2024-07-18

**Authors:** Hayley M. Belli, Chris S. Bresee, Matthew M. Graff, Mitra J. Z. Hartmann

In Fig 9, the location of datapoints for the incisors and dorsal ears are incorrect. Additionally, some of the shaded cells contain incorrect numerical values. Please see the correct Fig 9 here.

**Fig 9:**
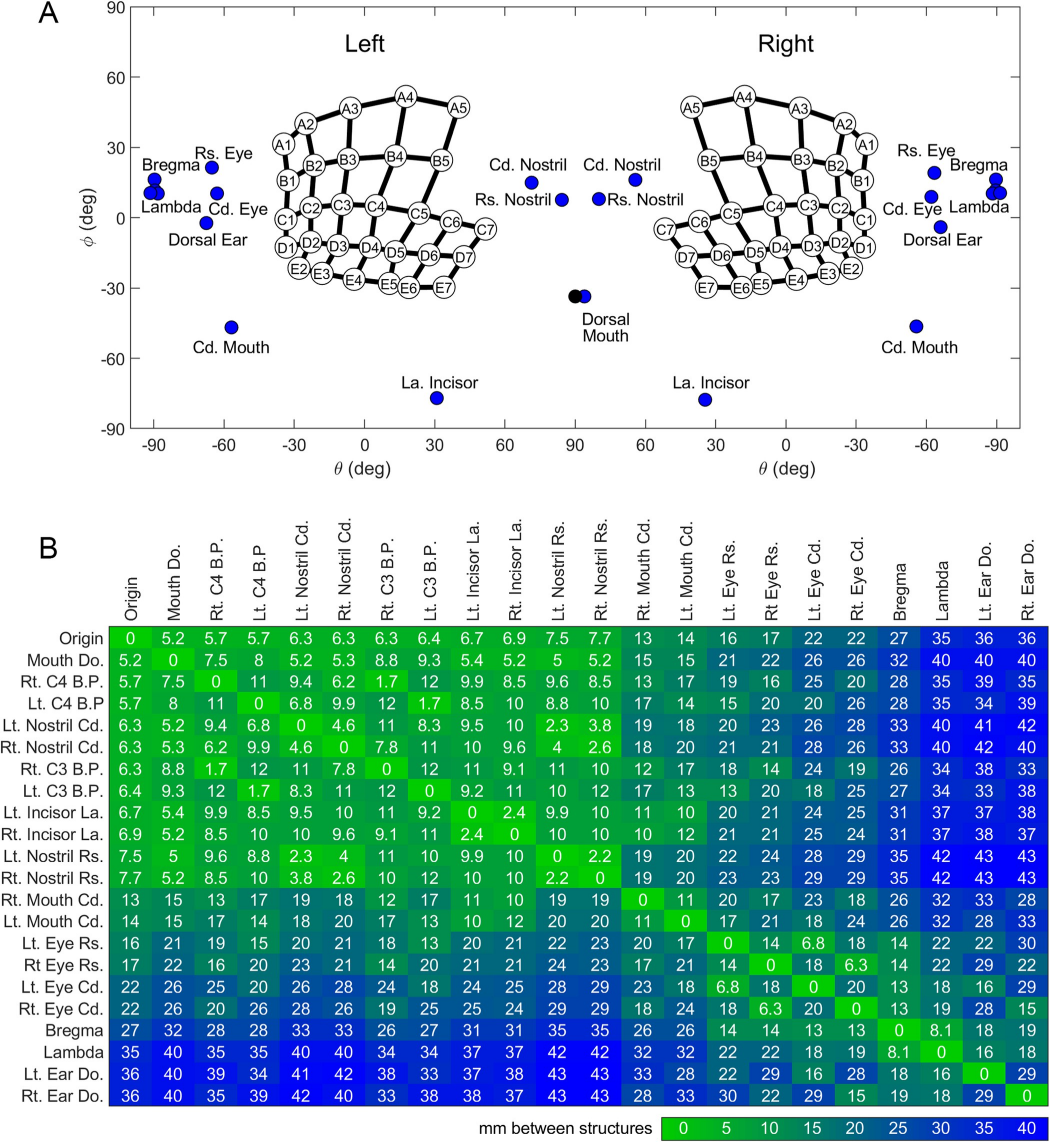
Quantification of coordinates of whisker basepoints, skull and facial features, and distances between these structures. (A) Position of the eyes, pinnae, nostrils, mouth, incisors, and bregma and lambda on the rat using coordinates θ and φ. The whisker array has been aligned into standard position and orientation using the average row plane. θ and φ are measured from the origin representing the average of all matched left and right whisker basepoints. The black dot indicates the theoretical dorsal mouth location. Notice that in this figure, the right and left facial features were not averaged. The left [right] facial features represent the average of the left [right] sides of five rats. (B) Average straight-line distances (mm) between facial features. Green indicates smaller distances, while blue indicates larger magnitudes. Entries in the array are sorted by proximity to the origin. Abbreviations: Lt. = left, Rt. = right, B.P. = basepoint, Cd. = caudal, Rs. = rostral, Do. = dorsal, La. = lateral.

In Fig 10, the location of datapoints for the incisors and dorsal ears are incorrect. Please see the correct Fig 10 here.

**Fig 1:**
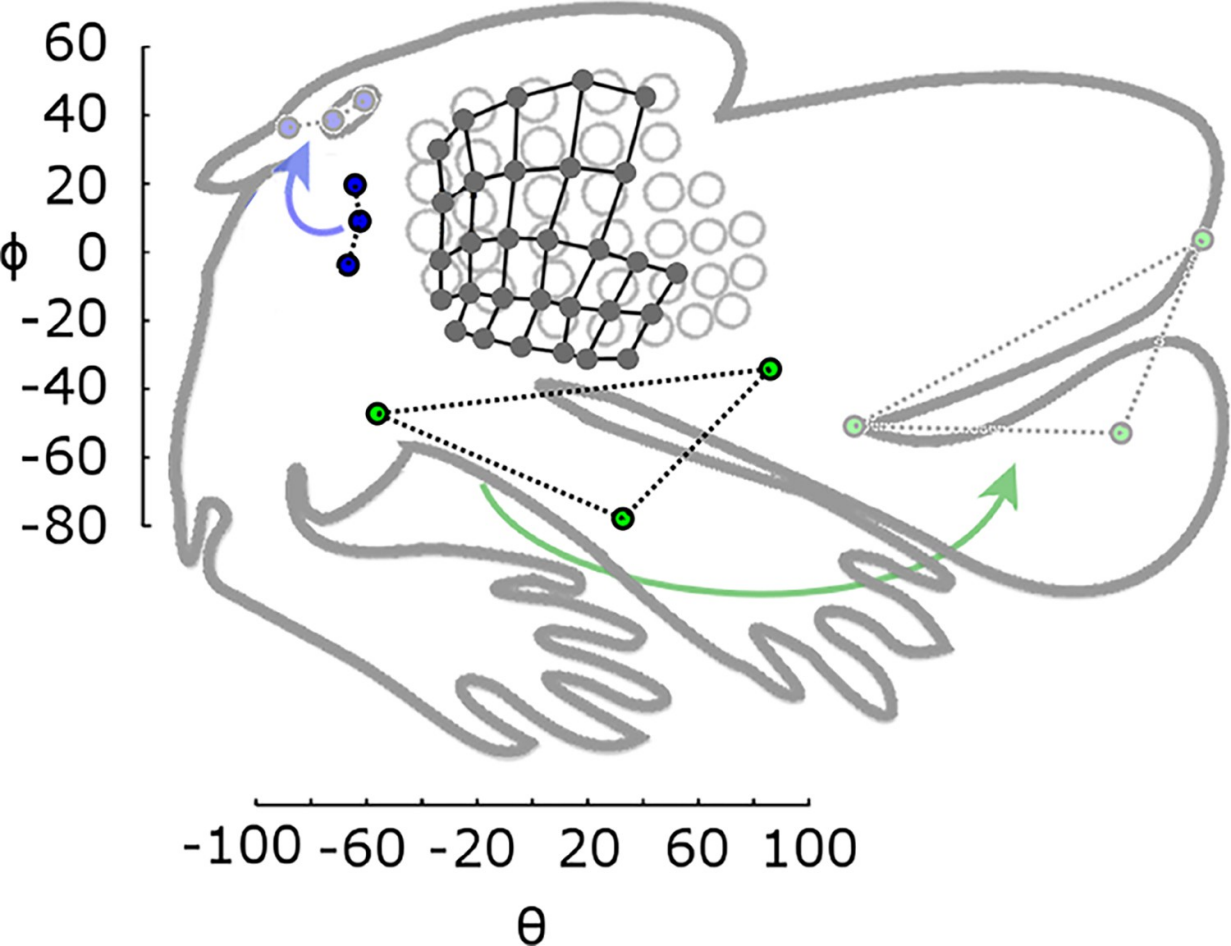
Proportion of angular area of facial features corresponds with proportion of cortical area. The ratunculus (grey outline, adapted from [1]) is rotated and scaled to approximately align the barrel representations (grey circles) with the angular locations of the basepoints from the present study (black circles connected by black grid lines). Blue points represent angular locations of the rostral and caudal points of the eye, and the dorsal corner of the pinna. When these are translated and rotated (but not scaled) they align with the features on the ratunculus (light blue circles). Similarly, the green points, representing the rostral and caudal corners of the mouth and the incisors, align with those features after repositioning (but not scaling). The nose shows a similar pattern but is not shown for visual clarity.

Supplementary Table 5 contains some incorrect values. Please view the correct Supplementary Table 5 below.

## Supporting information

S1 Dataset Data to make Fig 9. (XLSX)

## References

[1] Belli HM, Bresee CS, Graff MM, Hartmann MJZ (2018) Quantifying the three-dimensional facial morphology of the laboratory rat with a focus on the vibrissae. PLOS ONE 13(4): e0194981. doi: 10.1371/journal.pone.0194981 29621356 PMC5886528

