# Supplementary figures and images for: Quantifying the three-dimensional facial morphology of the laboratory rat with a focus on the vibrissae
Source: PLoS One. 2018 Apr 5;13(4):e0194981. doi: 10.1371/journal.pone.0194981 (PMC5886528; doi:10.1371/journal.pone.0194981)

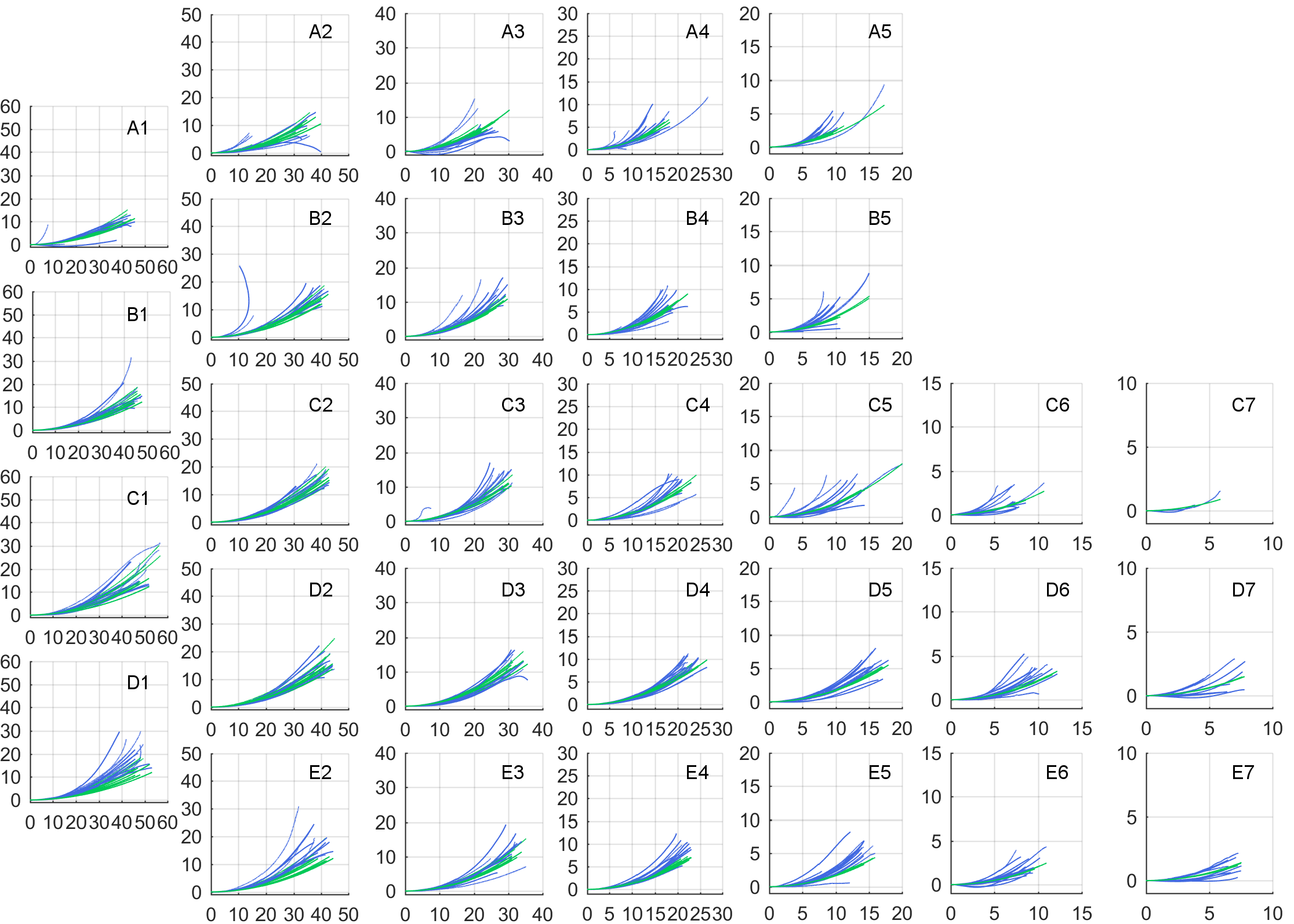

Supplement: S1 Fig — In all subplots, both axes have units of millimeters. Axes are square and equal so that the whisker aspect ratio is depicted accurately. Each subplot shows traces for a different whisker identity (row and column). Blue traces illustrate the scanned whiskers, smoothed and oriented to align with the x-axis as described in Methods. Green traces represent the fit for each whisker based on Eq 5 from the main text. To create each equation-based whisker, x values were obtained from each experimentally-measured whisker and the equation y = Ax2 was then plotted. There are exactly as many green traces (equation-based whiskers) in each subplot as there are blue traces (experimentally-measured whiskers). However, there appear to be fewer green traces because they overlap each other a great deal. The overlap occurs because θbp is very similar for all whiskers with a given row and column identity. This figure illustrates that Eq 5 captures the approximate shape of the whiskers, but cannot capture the high variability in whisker curvature, especially for the more rostral whiskers. Intrinsic whisker curvature is bounded by a strict upper threshold (Fig 6E), but exhibits high variability below that threshold. (TIF) [file pone.0194981.s001.tif]
