# Supplementary material for: Quantifying the three-dimensional facial morphology of the laboratory rat with a focus on the vibrissae
Source: PLoS One. 2018 Apr 5;13(4):e0194981. doi: 10.1371/journal.pone.0194981 (PMC5886528; doi:10.1371/journal.pone.0194981)
Supplement: S1 Table — (PDF) [file pone.0194981.s002.pdf]

Whisker ID	r_{bp} (mm)	θ_{bp} (degrees)	φ_{bp} (degrees)
A1	7.73	-35.2	31.4
A2	6.73	-25.0	39.5
A3	6.06	-5.98	45.8
A4	5.95	17.6	50.9
A5	5.91	40.2	47.1
B1	7.80	-32.6	15.9
B2	6.84	-22.2	21.9
B3	6.03	-7.83	25.0
B4	5.46	12.4	26.3
B5	5.45	31.9	23.7
C1	8.46	-33.6	-0.677
C2	7.38	-22.9	3.95
C3	6.42	-8.63	5.66
C4	5.76	4.92	5.39
C5	5.56	23.3	1.60
C6	5.82	36.9	-1.63
C7	6.45	51.6	-5.50
D1	8.84	-33.5	-12.6
D2	7.77	-23.0	-9.79
D3	7.04	-12.2	-11.9
D4	6.36	1.63	-12.0
D5	6.12	13.2	-14.1
D6	6.31	28.5	-15.8
D7	6.30	42.5	-16.7
E2	8.42	-28.0	-21.6
E3	8.05	-16.8	-23.8
E4	7.33	-4.48	-26.3
E5	6.96	6.91	-28.0
E6	6.77	20.7	-29.2
E7	6.74	33.9	-29.7

S1 Table. Average r_{bp}, θ_{bp}, and φ_{bp} by whisker identity (ID)
