# Supplementary material for: Quantifying the three-dimensional facial morphology of the laboratory rat with a focus on the vibrissae
Source: PLoS One. 2018 Apr 5;13(4):e0194981. doi: 10.1371/journal.pone.0194981 (PMC5886528; doi:10.1371/journal.pone.0194981)
Supplement: S1 Appendix — (PDF) [file pone.0194981.s003.pdf]

S1 Appendix. Equations as functions of row and column position

The present work uses basepoint parameters (θ_{bp} and φ_{bp}) as fundamental parameters to describe the geometry of the array, but previous work has used row and column identity. For completeness, this appendix quantifies the relationships for r_{bp}, S, A, θ_w, φ_w, and ζ_w using row and column identity as predictors. All of these equations were formed using the same number of whiskers as Eqs 3-9 in *Results*. In the following equations, Row varies from 1 to 5 and Col from 1 to 7:

$$r_{bp} = -1.31\ Col + 0.127\ Col^2 + 9.46, Adj. R^2 = 0.51$$

$$S = e^{-0.344\ Col + 4.36}, Adj. R^2 = 0.86$$

$$A = 0.00317\ Col + 0.00333, Adj. R^2 = 0.30$$

$$\theta_w = 2.73\ Row + 8.21\ Col + 28.0, Adj. R^2 = 0.54$$

$$\varphi_w = -17.8\ Row + 65.6, Adj. R^2 = 0.78$$

$$\zeta_w{}^* = 16.3\ Col - 18.5\ Row + 40.5, Adj. R^2 = 0.50$$

* Valid only for whiskers with arc length (S) >= 8 mm

For comparison, we repeat the equations from the main text in terms of basepoint parameters (θ_{bp} and φ_{bp}):

$$r_{bp} = 0.000511\ \theta_{bp}{}^2 - 0.0295\ \theta_{bp} - 0.0162\ \varphi_{bp} + 6.50, Adj. R^2 = 0.65 \quad (3)$$

$$S = e^{-0.0246\theta_{bp} + 3.12}, Adj. R^2 = 0.85 \quad (4a)$$

$$A = 0.000240\ \theta_{bp} + 0.0148, Adj. R^2 = 0.34 \quad (5)$$

$$\theta_w = 0.598\ \theta_{bp} - 0.314\ \varphi_{bp} + 67.4, Adj. R^2 = 0.64 \quad (7)$$

$$\varphi_w = 1.04\ \varphi_{bp} + 6.68, Adj. R^2 = 0.85 \quad (8)$$

$$\zeta_w{}^* = 0.876\ \theta_{bp} + 0.845\ \varphi_{bp} + 37.9, Adj. R^2 = 0.42 \quad (9)$$

* Valid only for whiskers with arc length (S) >= 8 mm

Comparing these two sets of equations yields the following observations:

- The variable r_{bp} is better predicted by basepoint coordinates than by row and column identity, as there is a large difference in the adjusted R^2 values (0.65 vs. 0.51).

- The variable S is equally well fit by basepoint coordinates as by row and column identity, as there is little difference between the adjusted R^2 values (0.85 vs. 0.86).

- Although predicting the intrinsic curvature coefficient A using basepoint parameters rather than row and column identity yields a slightly higher adjusted R^2 value (0.34 vs. 0.30), the quality of

prediction is poor for both equations. As discussed in *Results*, quantifying the upper bound of A as a function of S may serve as a better indicator of the wide range of values for A.

- The variable θ_w is slightly better predicted using θ_{bp} and φ_{bp} rather than row and column identity, exhibiting an improvement in adjusted R^2 by about 0.1. Both equations indicate that θ_w is influenced by changes to the whisker basepoint location in both the rostral-caudal and dorsal-ventral directions.

- The variable φ_w is slightly better predicted using φ_{bp} rather than row identity, exhibiting an improvement in adjusted R^2 by about 0.07. Both equations indicate that φ_w is only statistically influenced by changes in the dorsal-ventral location of the whisker basepoints.

- The angle ζ_w is the only parameter to be better described as a function of row and column identity than basepoint parameters, but the quality of the fit for both equations is poor.
